# A Dynamic Optimization Algorithm Based on Energy Level Collaboration Mechanism

**DOI:** 10.3390/e28070818

**Published:** 2026-07-17

**Authors:** Quan Tang, Yazhi Yang, Jing Liu

**Affiliations:** School of Computer Engineering, Chengdu Technological University, Chengdu 611730, China; tangquan20@mails.ucas.ac.cn (Q.T.);

**Keywords:** optimization problems, multi-population strategy, comprehensive learning, global exploration capability, adaptive migration

## Abstract

Complex multimodal optimization problems are widespread in machine learning, engineering design, and data science, where multiple local optima often trap conventional algorithms. Balancing global exploration and local exploitation remains a fundamental challenge for population-based optimization algorithms when solving such problems. This paper proposes a dynamic search framework optimization algorithm based on an energy level collaboration mechanism, termed DSF-ELC. The algorithm introduces two synergistic strategies. First, a population dynamic reorganization strategy adaptively adjusts particle migration between two fitness-stratified subpopulations based on real-time diversity measurements, effectively balancing exploration and exploitation. Second, a comprehensive learning strategy enables each dimension of inferior solutions to learn from the corresponding dimension of superior solutions in a randomized manner, thereby enhancing search capability on complex multimodal functions. The two strategies work synergistically to achieve an adaptive exploration-exploitation balance. Experimental validation on the CEC 2017 benchmark suite demonstrates that DSF-ELC achieves superior solution accuracy and stability compared to six representative algorithms on the vast majority of functions. Wilcoxon signed-rank tests, box plot visualization, and convergence curve analysis further validate the effectiveness of the proposed strategies. The results indicate that DSF-ELC has significant advantages and broad application prospects for complex multimodal optimization problems.

## 1. Introduction

Complex optimization problems widely exist in machine learning, engineering design, data science, and many other fields. As the number of decision variables increases, the solution space expands exponentially, making it extremely challenging to balance global exploration and local exploitation [1,2]. Serani and Diez [3] conducted a comprehensive survey on design-space dimensionality reduction methods, highlighting that the exponential growth of solution space remains one of the most fundamental challenges in optimization. This phenomenon requires optimization algorithms to possess strong global search capabilities to avoid local optima while maintaining sufficient local exploitation accuracy to approach the global optimum.

Population-based evolutionary algorithms and swarm intelligence algorithms have been extensively studied to address these challenges. Since Holland introduced the genetic algorithm (GA) in the 1970s [4], numerous classic algorithms have emerged, including particle swarm optimization (PSO) [5], differential evolution (DE) [6], and artificial bee colony (ABC) [7]. Among these, the Comprehensive Learning Particle Swarm Optimizer (CLPSO) proposed by Liang et al. [8] introduced a comprehensive learning strategy that enables each dimension of a particle to learn from different exemplars, effectively preventing premature convergence. However, CLPSO still suffers from insufficient local exploitation capability on multimodal and hybrid functions.

Recent advances have focused on adaptive parameter control and population diversity maintenance. Success-history-based adaptive differential evolution (SHADE) [9] and its variants have demonstrated competitive performance on the CEC benchmark suites. Multi-population strategies have also gained attention, as they naturally maintain diversity through subpopulation interactions. Research on migration processes in multi-population evolutionary models has revealed that classical migration guidelines from two decades ago may not be suitable for today’s complex optimization problems [10]. Dynamic multi-swarm strategies [11,12] and autonomous population size adjustment methods [13] have shown promise in maintaining diversity without manual parameter tuning.

However, existing multi-population methods typically use fixed migration policies that do not adapt to real-time population states. Furthermore, most dimension-wise learning mechanisms are treated as static strategies rather than being adaptively coordinated with population-level structures. The challenge of adaptively balancing exploration and exploitation on complex multimodal problems remains an ongoing research priority.

Inspired by the limitations of existing methods, this paper proposes a dynamic search framework based on an energy level collaboration mechanism (DSF-ELC). The algorithm introduces two novel mechanisms to enhance optimization performance on complex multimodal problems. First, a population dynamic reorganization strategy based on a two-level fitness stratification model dynamically adjusts particle migration between subpopulations using an adaptive probability. Second, a dimension-wise comprehensive learning strategy enables each dimension of inferior solutions to independently learn from superior solutions, complementing the global search capability of the multi-level structure. These two strategies work synergistically to achieve an adaptive balance between exploration and exploitation.

The main contributions of this paper are as follows:

(1) Proposing a population dynamic reorganization strategy based on a multi-level fitness stratification model, where migration probabilities adaptively adjust according to population diversity.

(2) Using a two-level model to design a comprehensive learning strategy that performs dimension-wise learning from relatively superior solutions to enhance global exploration capabilities.

(3) Extensive experiments on the CEC 2017 benchmark suite at dimensions 10 and 30 demonstrate that DSF-ELC achieves competitive solution accuracy and stability compared to six representative optimization algorithms.

(4) Parameter sensitivity analysis and ablation studies are conducted to validate the effectiveness of each proposed component.

The remainder of this paper is organized as follows. Section 2 reviews related work. Section 3 presents the proposed DSF-ELC algorithm. Section 4 provides a theoretical analysis. Section 5 reports experimental results. Section 6 concludes the paper.

## 2. Related Work

Complex multimodal optimization problems face a fundamental challenge: balancing global exploration to avoid local optima and local exploitation to achieve high-precision solutions. This section reviews two aspects of research that address this challenge: multi-population strategies to maintain a basic balance between exploration and exploitation, comprehensive dimensional learning strategy for enhancing the exploration of complex multimodal functions within a certain range. The limitations of existing methods motivate the design of the proposed DSF-ELC.

Population dynamic reorganization strategy for balancing global exploration and local exploitation. Maintaining population diversity is essential for effective global exploration of complex multimodal landscapes. A widely adopted approach is the use of multiple subpopulations, where individuals evolve independently and occasionally exchange information through migration. A self-adaptive multi-group particle swarm optimization algorithm is proposed [14], which adaptively divides a swarm into several sub-swarms and uses a competitive mechanism to select samples, and it helps the algorithm achieve an appropriate balance between exploration and exploitation. And the group maintains the ability for continuous exploitation. Ren et al. [15] demonstrated that multi-subpopulation competition strategies can effectively maintain population diversity while enabling independent evolution across subpopulations. Ding et al. [16] proposed an adaptive migration strategy within a multi-population co-evolutionary framework, demonstrating that appropriate resource allocation and information exchange between subpopulations can sustain population diversity and improve global exploration. For multi-objective optimization, a two-stage coevolutionary algorithm based on adaptive weights (AW-TCEA) [17] has been proposed to balance convergence, diversity and feasibility to handle constrained multi-objective problems (CMOPs) with complex Pareto fronts (PFs). Despite their effectiveness in maintaining diversity, most existing multi-population methods employ fixed migration policies that do not adapt to real-time population states. Based on this, we propose the first strategy: a population dynamic reorganization strategy that adaptively adjusts migration probability based on population diversity to balance global exploration and local exploitation.

Comprehensive learning strategy for enhanced exploration. Although multiple swarm strategies have maintained a certain balance between global exploration and local exploitation, stronger global exploration capabilities are often required for later exploitation of complex multimodal functions to break out of local optima. Dimension-wise learning mechanisms address this limitation by allowing individuals to learn independently across different dimensions. The comprehensive learning (CL) strategy, originally proposed in CLPSO [8], allows each dimension of a particle to learn from the corresponding dimension of different exemplars, including the particle’s personal best and the global best. This dimension-wise approach prevents premature convergence by preserving diversity across dimensions, as each dimension can follow a different learning trajectory. Building upon this concept, a two-swarm learning PSO (TSLPSO) algorithm based on different learning strategies is proposed to further improve global optimization performance [18]. Xie et al. [19] extended this idea to domain-dimensional learning, where guiding particles are generated based on the average of current particle dimensions, achieving a better balance between local and global search through hybrid mutation strategies. However, most existing dimension-wise learning mechanisms are treated as static strategies operating within a single population, without coordination with population-level structures. Based on this, we propose the second strategy: a comprehensive learning strategy with the multi-population framework established by the first strategy. While the proposed population dynamic reorganization strategy provides a basic balance between exploration and exploitation, its capability for highly multimodal and hybrid functions remains limited. Integrating comprehensive learning into the multi-population framework to strengthen global exploration of complex multimodal functions. By allowing each dimension of inferior individuals to learn from superior solutions across different dimensions, the strategy effectively helps the algorithm escape local optima and maintain search diversity.

In summary, existing multi-population strategies excel at exploration but often lack adaptive migration mechanisms. Existing dimension-wise learning strategies enhance exploitation but are rarely coordinated with population-level structures. The proposed DSF-ELC addresses these gaps by:

(1) Introducing an adaptive fitness-level probability that dynamically adjusts migration between multiple subpopulations based on real-time population monitoring.

(2) Integrating dimension-wise comprehensive learning with the multi-level structure in a synergistic manner to prevent populations from stagnating in multimodal landscapes.

## 3. DSF-ELC Overall Framework

### 3.1. Classical Dynamic Search Framework

The proposed DSF-ELC algorithm is developed under a classical dynamic search framework (DSF). As a general gradient-free population-based optimization paradigm, DSF conducts iterative search using a set of candidate solutions. Each iteration consists of three standard operations: solution sampling from the current population, fitness evaluation using the objective function, and selection of superior individuals for the next generation. All updating rules and evolutionary operations strictly follow the principles of classical evolutionary computation and stochastic search.

The pseudocode of DSF is shown in Algorithm 1. The initial search range is UB−LB, *q* is the particle number, $\sigma_{\min}$ is the accuracy of the optimal solution, the initial sampling scale $\sigma_{s}=UB-LB$, and max FE is the maximum number of iterations. $x^{\mathrm{mean}}$ is the average of $x_{i}$: $x^{\mathrm{mean}}=\mathrm{mean}\left(x_{i}\right)$. $x^{\mathrm{worst}}$ is the worst solution of $x_{i}$: $f\left(x^{\mathrm{worst}}\right)=\mathrm{argmax}\left(f\left(x_{i}\right)\right)$.
**Algorithm 1:** DSF algorithm pseudocode  **1**  **Input**: q, $\sigma_{\min}$, $\sigma_{s}=UB-LB$, randomly generated $x_{i}\left(i=1,2,\ldots ,q\right)$ in the domain [LB,UB]  **2**  **while** ($\sigma_{s}>\sigma_{\min}$) or (ite<max FE) **do**  **3**          **while** ($\sigma_{q}>\sigma_{s}$) **do**  **4**                   set $Flag_{\mathrm{stable}}=0$  **5**                   **while** ($Flag_{\mathrm{stable}}==0$) **do**  **6**                           $Flag_{\mathrm{stable}}=1$  **7**                           $\forall x_{i}$, generate $x_{i}^{\prime }∼N\left(x_{i},\sigma_{s}^{2}\right)$    (Normal sampling)  **8**                           **if** $f\left(x_{i}^{\prime }\right)<f\left(x_{i}\right)$  **9**                                  $x_{i}=x_{i}^{\prime }$    (Replace inferior solutions with optimal solutions)**10**                           **end if****11**                   **end while****12**                   Update the worst sampling particle: $x^{\mathrm{worst}}=x^{\mathrm{mean}}$    (Mean replacement)**13**                   Calculate the standard deviation $\sigma_{q}$ of all sampled particles $x_{i}$**14**          **end while****15**          $\sigma_{s}=\sigma_{s}/C_{r}$    (Sampling scale reduction)**16**  **end while****17**  **output**: $x^{\mathrm{best}}$, $f(x^{\mathrm{best}})$

DSF consists of two stages:

(1) Optimize the system to evolve into the optimal solution region at the current scale. It is mainly achieved through two layers of internal loops. At the current sampling scale, a large number of particles iterate continuously and eventually converge to the ground state. The innermost loop includes operations such as normal sampling, optimal solution acceptance, and mean replacement of particles at the current sampling scale.

(2) Multi-scale processes. It is mainly implemented through external loops. The external loop controls the operation of the entire iteration process and gradually reduces the sampling scale. The termination condition is that the optimal solution achieves accuracy or the algorithm reaches the maximum number of iterations.

Notably, DSF provides a stable, theoretically rigorous, and easy-to-implement foundation for designing advanced strategies such as population scheduling and dimension-wise learning. On the basis of the above classical dynamic search framework, this paper further proposes two core strategies:

(1) Population dynamic reorganization strategy based on energy level probability, which realizes adaptive subpopulation division and individual migration to enhance global exploration.

(2) Comprehensive dimension-wise learning strategy, which enables each dimension to learn from superior individuals randomly to improve local exploitation accuracy.

These two strategies are embedded in the DSF to form the final DSF-ELC algorithm.

### 3.2. Population Dynamic Reorganization Strategy

#### 3.2.1. Multilevel System

**Figure 1:**
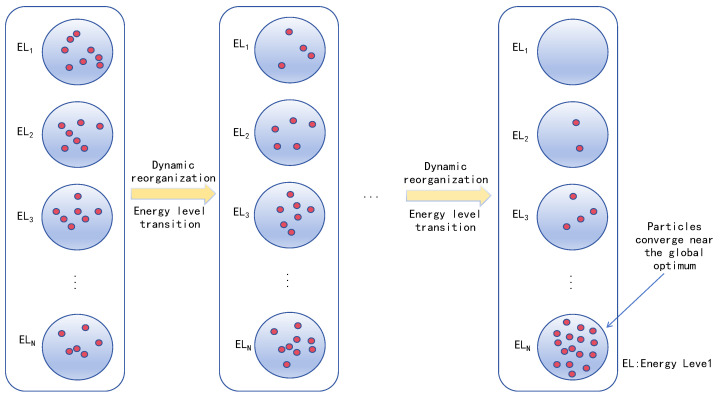
Schematic diagram of population reorganization.

Influenced by the fitness values of the objective function, the sampled particles migrate according to the rules defined by the optimization algorithm to explore the entire solution space. In DSF, multiple populations are dynamically reorganized based on the fitness values of the sampled particles. Each population corresponds to an energy level, and each energy level contains multiple sampled particles. “Energy level” is a metaphor for fitness ranking; high fitness corresponds to a high energy level, and low fitness corresponds to a low energy level. The particles obtain new solutions through sampling, achieving energy level transitions. Multiple energy levels collaborate to complete the optimization iteration process, and ultimately, the particles converge near the global optimum, as shown in Figure 1.

In quantum physics, the energy levels of microscopic particles can be determined by calculating their energy, with the lowest energy level being the ground state. Correspondingly, in optimization algorithms, the energy of particles is determined by their fitness values, which, in turn, determine their energy levels. The higher the fitness value, the higher the energy level, and vice versa.

#### 3.2.2. Strategy Based on Population Dynamic Reorganization

In statistical mechanics, the Boltzmann distribution represents the probability distribution or probability measure of particles in a system across various possible microscopic quantum states [20]. It takes the following form [21]:

(1) $$p_{i}=\frac{\exp\left(-\varepsilon_{i}/kT\right)}{\sum_{j=1}^{M}\exp\left(-\varepsilon_{j}/kT\right)}$$

where $p_{i}$ is the probability of quantum state *i*, $\varepsilon_{i}$ is the energy of quantum state *i*, *k* is the Boltzmann constant, *T* is the system temperature, and *M* is the number of quantum states possessed by the system.

The temperature *T* is a physical quantity that varies with time. Therefore, it can be seen that the probability of energy levels appearing over time in the optimization algorithm can be calculated using Equation (1). In optimization algorithms, sampling particles are assigned to different populations, with each population corresponding to a quantum state (quantized energy level).

Consider the quantum state probability in Equation (1) as the proportion of particles obtained by a population to the total number of particles in the population. Therefore, in the optimization algorithm, the proportion of particles obtained by the *i*th population $S_{i}$ to the total number of particles in the population is calculated using Equation (2):

(2) $$P\left(S_{i}\right)=\frac{\exp\left(-E_{i}\tau \right)}{\sum_{N}\exp\left(-E_{N}\tau \right)}$$

where *N* represents the population size. Correspondingly, the number of particles obtained by the *i*th population $S_{i}$ after each iteration is calculated according to Equation (3), as follows:

(3) $$m_{i}=P\left(S_{i}\right)\;\cdot \;m_{\mathrm{all}\;}$$

where $m_{i}$ represents the number of particles obtained by the *i*-th population, corresponding to the *i*-th energy level, and $m_{\mathrm{all}}$ denotes the total number of particles obtained by all populations. When there is a greater difference in the probabilities of population emergence, the population with a lower probability has a lower proportion and obtains fewer sampling particles. For certain functions with greater optimization difficulty, where local optima are scattered across numerous valleys, and the global optimum requires traversing multiple local optima, it is necessary to reduce the significant difference in the number of particles obtained by different populations due to the direct use of probabilities. This allows for obtaining relatively more sampling particles in regions with higher fitness values, enabling the algorithm to guide particles to escape from local optima. Therefore, the populations are sorted in descending order of their probabilities of emergence, satisfying $P\left(S_{r_{1}}\right)>P\left(S_{r_{2}}\right)>\ldots >P\left(S_{r_{N}}\right)$, where $r_{i}$ is the ranking of the population and $P(S_{r_{i}})$ represents the probability of the population ranked as $r_{i}$. The number of particles within each population is determined based on the population ranking $r_{i}$, as detailed in Equation (4).

(4) $$m_{i}=\frac{r_{i}}{{∥r_{i}∥}^{\beta }}\;\cdot \;m_{\mathrm{all}}$$

In this context, β serves as the sorting adjustment factor. A higher value of this factor leads to a greater disparity in the number of particles within the population. Directly using probability as the basis for determining the number of particles can result in an extremely uneven distribution of particles, making it difficult for them to fully explore the solution space. Therefore, a probability-based sorting approach is adopted to mitigate the abnormal disparities in particle numbers caused by the direct use of probability. The sorting factor β is designed to dynamically adjust the variability in the number of particles and facilitate controlled allocation of particle numbers. When β=1, the number of particles in the population is allocated according to a fixed gradient, forming an arithmetic sequence. As the value of β increases, there is an exponential difference in the number of particles within the population, with populations with lower probabilities obtaining fewer particles, or even none. Therefore, the value of β is generally not greater than 5 and is a positive integer. The subsequent experimental section will verify the selection of parameter β. The proportion of particle numbers within the population, calculated using Equation (4), is adjustable, and the number of particles in the population decreases at a fixed gradient, which is beneficial for reducing disparities among populations and enhancing the global search capability of the algorithm.

The process of the population reorganization strategy is shown in Algorithm 2.
**Algorithm 2:** Pseudo-code for population reorganization strategy**1**  **Input**: Population size *N*, total number of particles in the population $m_{\mathrm{all}}$, sorting adjustment factor β**2**  **for** i=1:N**3**          Calculate $P\left(S_{i}\right)$ using Equation (2)**4**          Sort $P\left(S_{i}\right)$ in descending order to obtain the population ranking $r_{i}$**5**          Calculate $m_{i}$ according to Equation (4)**6**  **end for****7**  **output**: The number of particles contained in each population, $m_{i}$

### 3.3. Comprehensive Learning Strategy

#### 3.3.1. Two-Level Approximation Determines Probability

When optimizing algorithms sample the objective function, there are usually only two outcomes: one is obtaining a better solution, and the other is obtaining an inferior solution. The information implied by the inferior solution can guide the optimization algorithm to perform a global search. For example, the simulated annealing algorithm adopts the Metropolis criterion to perform probabilistic acceptance of inferior solutions.

In quantum systems, the general solution of the Schrödinger equation can be written as follows:

(5) $$\psi \left(x,\tau \right)=\sum_{n}C_{n}\varphi_{n}\left(x\right)\exp\left(-E_{n}\tau \right)$$

where $C_{n}$ is the superposition coefficient. $E_{n}$ is the state energy, i.e., the quantized energy level.

According to Equation (5), the function values of two adjacent samples are represented by energies $E_{a}$ and $E_{b}$, respectively, where $E_{a}$ is the energy of the current solution and $E_{b}$ is the energy of the solution obtained from sampling. The superposition of energy states in Equation (5) is approximated as the superposition of two energy states, $E_{a}$ and $E_{b}$. Assuming the optimization problem is to find the minimum value, when an inferior solution is obtained, $E_{a}<E_{b}$, where $E_{a}$ is the low-energy state and $E_{b}$ is the high-energy state. Neglecting the reference energy $E_{R}$, Equation (5) is approximated as a two-level formula:

(6) $$\psi \left(x,\tau \right)=c_{a}\varphi_{a}\exp\left(-E_{a}\tau \right)+c_{b}\varphi_{b}\exp\left(-E_{b}\tau \right)$$

Among them, the inferior solution corresponds to the high-energy state, and the probability of the two energy levels occurring mainly depends on the exponential term. Therefore, the acceptance probability of the inferior solution can be approximated as follows:

(7) $$P_{b}\approx \frac{\exp\left(-E_{b}\tau \right)}{\exp\left(-E_{a}\tau \right)+\exp\left(-E_{b}\tau \right)}=\frac{1}{1+\exp(\Delta E\tau )}$$

where $\Delta E=E_{b}-E_{a}$, represents the energy level spacing, indicating the difference in fitness values between two consecutive samplings in the optimization algorithm. This formulation is consistent with classical simulated annealing.

Equation (7) represents the probability of accepting a bad solution based on the two-level approximation. When τ=0, the probability of accepting a bad solution, $P_{b}$, is 0.5. As time evolves and τ increases, the probability of accepting a bad solution, $P_{b}$, gradually decreases. In the iterative process of the optimization algorithm, time is characterized by the number of algorithm iterations. In this algorithm, τ is reset to 0 at each iteration scale and gradually increases with the iteration process. Furthermore, τ is positively correlated with the number of evolutionary steps $t_{\mathrm{evol}}^{i}$ at the *i*th scale. Let $\tau =\alpha \;\cdot \;t_{\mathrm{evol}}^{i}$, where α is a contraction-expansion factor that adjusts the probability of accepting a bad solution, $P_{b}$, to ensure optimization performance. Substituting this into Equation (7) yields Equation (8):

(8) $$P_{b}\approx \frac{1}{1+\exp\left(\Delta E\;\cdot \;\alpha \;\cdot \;t_{\mathrm{evol}}^{i}\right)}$$

In Equation (8), although there is a positive correlation between time τ and the number of evolution times $t_{\mathrm{evol}}^{i}$, due to the complexity of the algorithm’s search process, the final impact on the probability of learning inferior solutions $P_{b}$ still requires parameter experimentation on α to select the optimal parameter.

#### 3.3.2. Strategy Based on Comprehensive Learning

When a particle sampling yields an inferior solution, it should be discarded according to the usual rules. This approach tends to prematurely trap in local optima for complex function optimization. Therefore, when a particle sampling yields an inferior solution, a comprehensive learning strategy is adopted to transform and learn the current particle from all dimensions with a certain probability, helping the particle escape from local optima. When the *k*th particle $x_{k}$ in the current population yields an inferior solution, the comprehensive learning strategy is initiated according to the probability calculated by Equation (8). For complex functions, changes in each dimension may cause significant differences in the fitness value, so the comprehensive learning strategy needs to learn and update each dimension of the particle.

**Figure 2:**
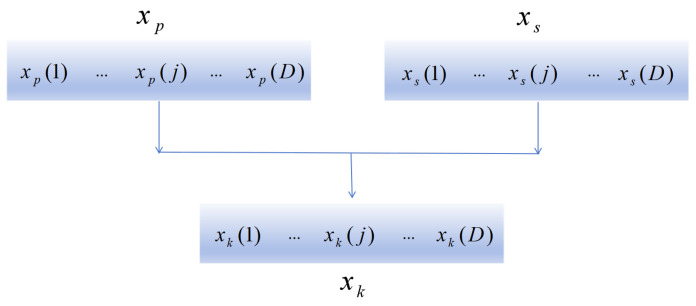
Schematic diagram of comprehensive learning strategy.

As shown in Figure 2, the main process of the comprehensive learning strategy is as follows: Randomly select two particles, $x_{p}$ and $x_{s}$, from the current population for learning. Assuming the dimension of the optimization problem is *D*, then $x_{p}\left(j\right)$ represents the *j*th dimension of particle $x_{p}$. For any dimension *j* of the sampled particles, generate a random number ${\mathrm{rand}}_{j}$, respectively. When ${\mathrm{rand}}_{j}$ is less than the probability $P_{b}$ of comprehensive learning for inferior solutions obtained from Equation (8), compare the fitness values $f(x_{p})$ and $f(x_{s})$ of the two particles, and select the particle with superior fitness to replace its *j*th dimension with the corresponding dimension of $x_{k}$.

The process of the comprehensive learning strategy is shown in Algorithm 3.
**Algorithm 3:** Pseudo-code for comprehensive learning strategy  **1**  **Input**: Particle $x_{k}$, optimization problem dimension *D*, contraction-expansion factor α  **2**  **for** j=1:D  **3**        **if** ${\mathrm{rand}}_{j}<P_{b}$ (Calculate the comprehensive learning probability of inferior solutions through Equation (8))  **4**                Randomly select two particles $x_{p}$ and $x_{s}$ from the current population  **5**                **if** $f\left(x_{p}\right)<f\left(x_{s}\right)$  **6**                        $x_{k}\left(j\right)=x_{p}\left(j\right)$,  **7**                **else**  **8**                        $x_{k}\left(j\right)=x_{s}\left(j\right)$,  **9**                **end if****10**        **end if****11**  **end for****12**  **output**: The updated particle $x_{k}^{\prime }$

### 3.4. DSF-ELC Algorithm Flow

Based on the aforementioned population dynamic reorganization strategy and comprehensive learning strategy, the pseudo-code of the DSF-ELC algorithm is obtained, as shown in Algorithm 4.   
**Algorithm 4:** Pseudo-code for DSF-ELC algorithm **1**  **Input**: Population size *N*, total number of sampled particles $m_{\mathrm{all}}$, sorting adjustment factor β, contraction-expansion factor α, maximum iteration count max FE  **2**  **while** (ite<max FE) **do**  **3**          **while** (ite<max FE) **do**  **4**                   Set $Flag_{\mathrm{stable}}=0$  **5**                   **while** ($Flag_{\mathrm{stable}}==0$) **do** **6**                 Calculate the number of particles in each population according to Algorithm 2 (Population reorganization strategy)  **7**                           **for** i=1:N  **8**                              Obtain a new solution $x_{k}^{\prime }$ by normal sampling from the *k*-th particle in the *i*-th population, where $x_{k}^{\prime }\;N\left(x_{k},\sigma_{s}^{2}\right)$  **9**                             **if** $f\left(x_{k}^{\prime }\right)<f\left(x_{k}\right)$**10**                                   $x_{k}=x_{k}^{\prime }$, $Flag_{\mathrm{stable}}=0$**11**                             **else****12**                                   Update particles according to Algorithm 3 (Comprehensive learning strategy)**13**                             **end if****14**                           **end for****15**                   **end while****16**                   Update the worst sampled particle: $x^{\mathrm{worst}}=x^{\mathrm{mean}}$**17**                   Calculate the standard deviation of all sampled particles, $\sigma_{\mathrm{all}}$**18**                   **if**($\sigma_{\mathrm{all}}<\sigma_{s}$)**19**                           **break****20**                   **end if****21**          **end while****22**          $\sigma_{s}=\sigma_{s}/C_{r}$**23**  **end while****24**  **output**: Optimal solution $x^{\mathrm{best}}$ and optimal fitness value $f(x^{\mathrm{best}})$

In Algorithm 4, $C_{r}$ is the scale reduction coefficient, dynamically adjusting the scale contraction speed.

### 3.5. Interpretation of Exploration-Exploitation Balance

The population reorganization strategy introduces an adaptive migration probability based on population diversity. In the early stage of iteration, the differences in fitness values among subpopulations are small, and the number of particles obtained is comparable. At this time, the diversity is high, and migration will increase to introduce different individuals and promote exploration. In the later stage of iteration, particles gradually approach the global optimum, and the optimal subpopulation obtains a large number of particles. At this time, diversity is low, and migration is reduced to allow local exploitation.

Although population reorganization strategies provide basic balance, getting rid of local optima on multimodal functions requires stronger global exploration. Add comprehensive learning strategies that allow learning from each dimension of the optimal solution sample for the inferior solution. This helps the algorithm avoid local optima and maintain search diversity. The learning probability Pb decreases from 0.5 to nearly 0 during the iteration process. In the early stages, a higher Pb encourages learning (exploration) from different examples. In the later stage, with lower Pb, the focus shifts to the global optimum (exploitation) to accelerate convergence.

## 4. Theoretical Analysis of DSF-ELC

### 4.1. Time Complexity Analysis

The time complexity of DSF-ELC is mainly determined by the main iteration loop, population dynamic reorganization, and comprehensive learning strategy. Let *N* represent the population size, *D* represent the problem dimension, and max FE represent the maximum number of iterations. The population restructuring strategy involves fitness calculation and ranking, with a complexity of O(N×logN). In the worst case, the comprehensive learning strategy updates the dimensions of each individual, resulting in a complexity of O(N×D) for each iteration. Compared with O(N×D), O(N×logN) can be ignored, so the overall time complexity of DSF-ELC is O(max FE×N×D), which is linearly related to the overall size and dimension. This complexity is consistent with most population-based metaheuristic algorithms and is linearly related to the problem dimension *D*, indicating that DSF-ELC maintains good computational efficiency for complex multimodal optimization problems.

The time complexity of the proposed DSF-ELC was compared with six representative metaheuristic algorithms, including ABC, SPSO2011, SSA, dynFWA, LOTFWA, and CMAES. The time complexity of DSF-ELC is O(max FE×N×D), and it is linear in both population size and dimension. In contrast, due to the adaptability of the covariance matrix, CMAES exhibits a complexity of $O(\max\;FE\times N\times D^{2})$, resulting in significantly higher computational costs in the dimensionality of quadratic scaling and complex multimodal optimization problems. The fireworks algorithm variants dynFWA and LOTFWA have complexity O(max FE×(N+m)×D), where m is the number of sparks, of the same order of magnitude as N. ABC, SPSO2011 and SSA all have linear complexity O(max FE×N×D).

Comparison shows that DSF-ELC maintains the same linear complexity as SSA and SPSO2011, while being more effective than CMAES in high-dimensional scenarios. Compared with dynFWA and LOTFWA, DSF-ELC achieves better performance without increasing the additional overhead of spark generation and selection operations, demonstrating its good computational efficiency in complex optimization problems.

### 4.2. Convergence Analysis

#### 4.2.1. Convergence Analysis Based on Solis and Wets Conditions

Following the general random search convergence framework established by Solis and Wets [22], a stochastic optimization algorithm converges to the global optimum with probability 1 if it satisfies two sufficient conditions:

**Condition 1** Elite preservation: $f\left(x_{t+1}\right)\leq f\left(x_{t}\right)$, that is, the fitness value of the algorithm monotonically does not increase during the iteration process.

**Condition 2** Sampling coverage condition: For the global optimum and its small neighborhood in the search space, the probability of the algorithm sampling that neighborhood within a finite number of steps is strictly greater than 0.

Regarding **condition 1**, in the DSF-ELC algorithm, position update is only performed when the fitness of the newly sampled solution $x^{\prime }$ is strictly better than the current solution $x_{k}$, i.e., $f\left(x^{\prime }\right)<f\left(x_{k}\right)$. This ensures that the optimal fitness historical trajectory of the population is monotonically non-increasing, satisfying **condition 1**.

For **condition 2**, use a normal distribution to sample $x^{\prime }∼N\left(x_{k},\sigma \right)$. The probability density function of a normal distribution is strictly greater than 0 over the entire real number field $R^{D}$. In addition, the comprehensive learning strategy introduces dimensional information of other particles, further expanding the support set of sampling. As long as the scale reduction coefficient $C_{r}$ does not cause the standard deviation σ to go to zero within a finite number of steps, the probability of sampling covering the entire space is always greater than 0, satisfying **condition 2**.

Therefore, according to the Solis and Wets theorem, the DSF-ELC algorithm theoretically has global convergence, that is, it converges to the global optimal solution with a probability of 1.

#### 4.2.2. Convergence Analysis Based on Markov Chain

##### Basic Assumptions

To further analyze the convergence of DSF-ELC, we adopted a population-based metaheuristic Markov chain framework [23,24], which is widely accepted for heuristic optimization algorithms. This analysis is based on three assumptions:

(1) Finite state space. The search space is bounded, and the population state can be represented by a finite set of fitness vectors.

(2) Improved probability. For any non-optimal state, there exists a non-zero probability in the next iteration to generate a better solution.

(3) Elite preservation. The best solutions discovered so far are always preserved and will never be discarded.

These assumptions naturally apply to DSF-ELC, as it operates in a bounded continuous space with elite selection and random update rules.

##### Markov Chain Modeling and Global Convergence Proof

The population state during iteration *t* is defined as follows: $S\left(t\right)=\{x_{1}\left(t\right),x_{2}\left(t\right),\ldots ,x_{N}\left(t\right)\}$, and $S^{*}\left(t\right)$ represents a state that contains the global optimal value. Due to the fact that the transition probability P(S(t+1)∣S(t)) depends only on the current state and not on early history. And random operations follow fixed probability rules, such as sampling, reassembly, and dimension learning. Therefore, the update process of DSF-ELC forms a homogeneous Markov chain ${\left\{S\left(t\right)\right\}}_{t\geq 0}$.

The probability convergence of the DSF-ELC global optimum can be proven through the following two key steps.

Step 1: Irreversibility. There is a finite transition path for any two states $S_{a}$ and $S_{b}$, such that $P(S_{b}|S_{a})>0$. DSF-ELC satisfies this property:

(1) Population reorganization randomly reassigns individuals, making it possible for individuals to migrate between distant regions.

(2) The comprehensive learning strategy independently updates each dimension of the individual, ensuring that any coordinate can be modified with a non-zero probability.

(3) Gaussian sampling explores the neighborhood of each individual, covering the entire search space with a positive probability.

Therefore, there is no closed subset that can capture a population, and Markov chains are irreducible.

Step 2: Absorb the global optimal value. Set as the optimal state containing the global minimum value. According to the elite retention rule in DSF-ELC, once the global optimal value is found, it will always retain $P(S^{*}|S^{*})=1$. Therefore, it is an absorption state. For any non-optimal state *S*, irreducibility means that $P(S^{*}|S)>0$. By combining irreducibility and absorbing the optimal state, the Markov chain will eventually enter $S^{*}$ with a probability of 1 as t→∞.

#### 4.2.3. Impact of Core Strategies on Convergence

The population reorganization strategy dynamically allocates subpopulation sizes based on fitness ranking. The subpopulation with better fitness is allocated more particles $m_{i}$. This is essentially an adaptive selection pressure mechanism. It tilts computing resources towards promising and excellent search spaces, greatly accelerating the convergence process. But theoretically, if the selection pressure is too high, it may lead to premature loss of diversity.

When normal sampling fails, the comprehensive learning strategy utilizes information from other particles in the population for dimension-level cross-learning. Normal sampling has symmetry and is prone to falling into local symmetry traps, such as narrow canyon terrain. But the comprehensive learning strategy breaks this symmetry and provides powerful global exploration capabilities. It theoretically acts as a diversity maintainer, effectively preventing premature convergence of the algorithm and ensuring the actual performance of global convergence.

The worst particle update mechanism, i.e., $x_{\mathrm{worst}}=x_{\mathrm{mean}}$, pulls the particles with the worst fitness directly towards the center of the population mean. This introduces a strong centripetal force. In the later stage of the algorithm, when the population is close to the optimal solution, this operation can quickly remove free inferior particles, causing the population to quickly gather, thereby improving the convergence speed in the later stage.

Overall, these three complementary strategies enable DSF-ELC to dynamically balance exploration and exploitation, avoiding both premature convergence and insufficient local refinement, and ultimately ensuring superior optimization performance in complex multimodal optimization problems.

## 5. Experimental Design and Result Analysis

### 5.1. Parameter Test

#### 5.1.1. Parameter Sensitivity Analysis

Parameter selection is crucial for optimizing the performance of algorithms. Therefore, this section analyzes the parameter sensitivity of DSF-ELC. The experiment employs five test functions from CEC 2017 (multimodal function F8, hybrid functions F11 and F12, and composite functions F20 and F29) to conduct a full factorial experiment on three parameters of DSF-ELC (population size *N*, sorting adjustment factor β, and scaling factor α). The dimension D=10, and each function is run 30 times to obtain the mean value of the function fitness error. The three parameters are set to N={30,50,100}, β={2,3,4}, and α={0.1,1,10}, resulting in a total of 27 combinations.

**Figure 3:**
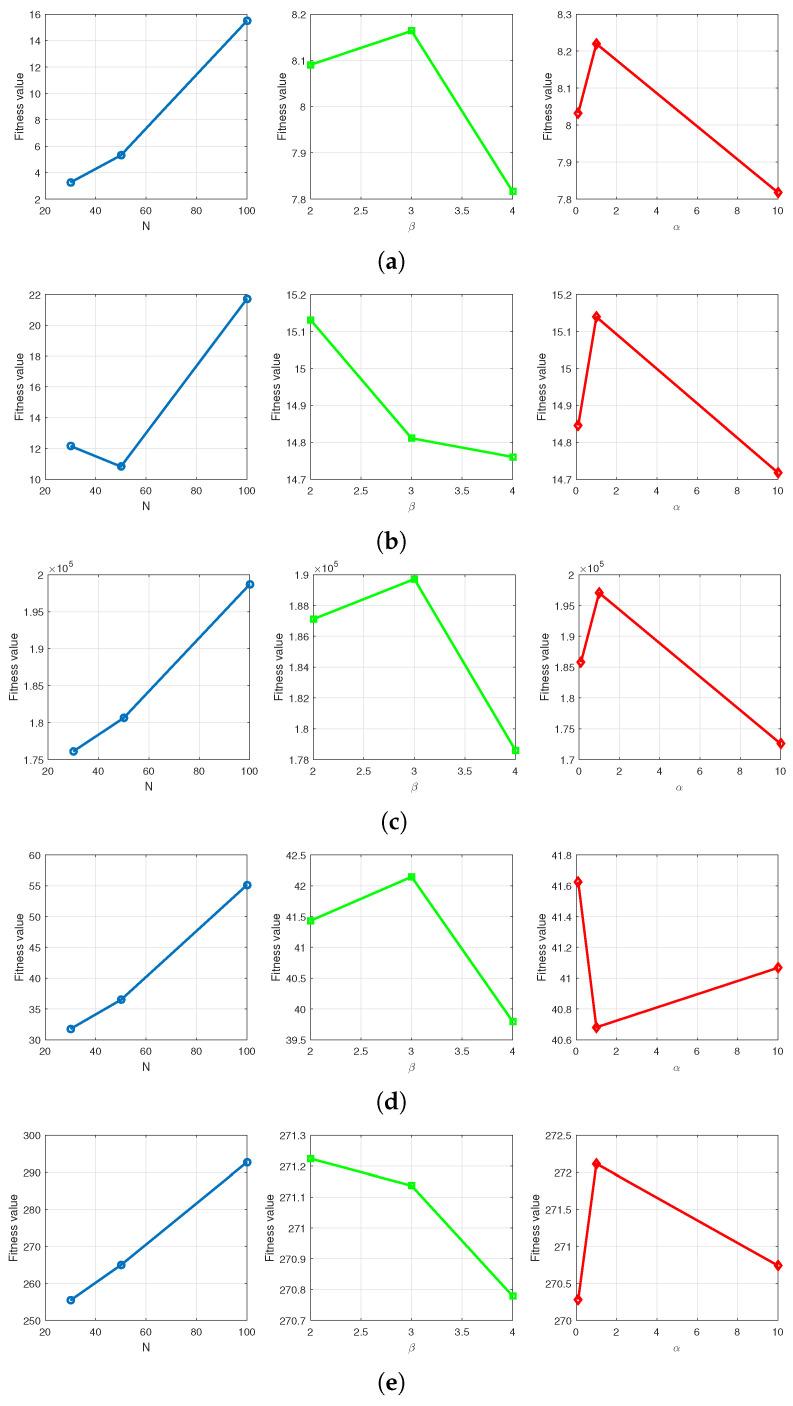
DSF-ELC algorithm’s parameter sensitivity experimental results: (**a**) F8. (**b**) F11. (**c**) F12. (**d**) F20. (**e**) F29.

As shown in Figure 3, the sensitivity test results of three parameters are presented. The *x*-axis represents the three control parameters, while the *y*-axis represents the mean of the fitness value error. For the five functions, the parameters *N*, β, and α all exhibit high sensitivity. For functions F8, F12, and F29, the sensitivity trends of the three parameters are roughly similar. Functions F8 and F12 perform optimally when N=30, β=4, and α=10. For function F29, it performs optimally when N=30 and β=4, with parameter α achieving optimality at 0.1. However, the mean error values for α=10 and α=0.1 are not significantly different. For function F11, consistent with other functions, optimal performance is achieved when β=4 and α=10. However, differently, parameter *N* achieves optimality at 30. For function F20, optimal performance is achieved when N=30 and β=4, consistent with other functions, but parameter α achieves optimality at 1. Overall, selecting N=30, β=4, and α=10 as the optimal parameter choices can achieve optimal fitness values for most functions.

#### 5.1.2. Population Size *N* and Sorting Adjustment Factor β

When testing the parameters population size *N* and sorting adjustment factor β, the scaling factor α is fixed at a certain value, which is set to α=10 in this experiment.

The performance comparison results of the DSF-ELC algorithm under different population sizes and sorting adjustment factors are shown in Table 1. When N=30,β={2,3,4}, the DSF-ELC algorithm achieves mean error rankings of 4.07, 3.47, and 3.40 on the 30 functions of the CEC 2017 test set, respectively, which are significantly better than the rankings of 6.70, 6.70, and 6.43 for N=100 and the rankings of 5.20, 4.77, and 4.27 for N=50. When N=30,β=4, the mean error rankings for multimodal and hybrid functions are 1.57 and 4.20, respectively, and the mean error ranking for all 30 functions is 3.40, with 8 optimal solutions, all ranking first. However, for unimodal functions, the combination N=30,β=4 is slightly weaker than the combination N=30,β=2, ranking second. For composite functions, the combination N=30,β=4 is weaker than the combinations N=30,β=2 and N=30,β=3, ranking third. In terms of stability, on the 30 test functions, the combination N=30,β=4 is weaker than the combinations N=30,β=2, N=30,β=3, and N=50,β=4, but significantly stronger than the other five combinations, ranking fourth. Considering the excellent performance of the combination N=30,β=4 in multimodal and hybrid functions, and considering both accuracy and stability, this combination is selected as the optimal parameter, at which the DSF-ELC algorithm achieves good performance. In the subsequent content of this paper, this parameter setting will be adopted.

#### 5.1.3. Scaling Factor α

In the comprehensive learning strategy, the probability of learning from inferior solutions, denoted as $P_{b}$, is obtained through Equation (8). It exhibits a nonlinear relationship with the scaling factor α. By adjusting the value of α, the probability of solution learning $P_{b}$ can be controlled, thereby achieving the goal of regulating population diversity. When α is smaller, the probability of learning from inferior solutions is higher, and the probability of updating each dimension of the inferior solutions is greater. This is suitable for conducting a comprehensive exploration of the solution space in the early stages of iteration to explore the global optimal region. Conversely, a high probability of learning from inferior solutions is suitable for exploiting the solution space in the middle and later stages of iteration to obtain a more precise optimal solution. However, due to the influence of other parameters, the value of α needs to be determined through parameter experiments. Section 5.1.2 has already determined the optimal values for population size and sorting adjustment factor: N=30,β=4. Therefore, the experimental parameters for this section are set as follows: dimension D=10, population size N=30, and sorting adjustment factor β=4.

The experimental data are presented in Table 2. α=10 ranks first in both the average rankings of F1–F3 and F21–F30, but its average rankings in F4–F10 and F11–F20 are slightly weaker than those of α=1 and α=0.1. However, considering the overall average ranking of the 30 functions, α=10 ranks first. In terms of stability, the standard deviations for the three different values of α are not significantly different. Regarding the solutions, the number of optimal solutions for α=10 is 12, which is significantly better than that of α=1 and slightly better than that of α=0.1. For functions F21 and F25, although the solution for α=10 is not optimal, it is very close to the optimal solution. Therefore, based on the above analysis, when α=10, DSF-ELC can achieve the overall optimal performance. Therefore, the parameter combination of N=30,β=4,α=10 is selected as the optimal parameter set, which is consistent with the conclusion of the parameter sensitivity test mentioned earlier. In the subsequent content of this paper, this parameter combination will be used for settings.

### 5.2. Ablation Study

To validate the contribution of each proposed component, we conduct an ablation study with three variants of DSF-ELC:

(1) DSF-ELC1: Comprehensive learning strategy disabled.

(2) DSF-ELC2: Population dynamic reorganization strategy disabled.

The full DSF-ELC is compared against these variants on the CEC 2017 benchmark suite at dimensions 10 and 30.

The parameter settings are shown in Table 3. The experiments were repeated 30 times each, using the CEC 2017 standard test suite. The experimental results for 10-dimensional and 30-dimensional problems are shown in Table 4 and Table 5, respectively.

As can be seen from Table 4, when the dimension D=10, DSF-ELC1 performs exceptionally well, far surpassing the other three algorithms, especially excelling in mixed functions and composite functions. This indicates that dynamic population restructuring significantly enhances the algorithm’s global optimization capability for complex functions. DSF-ELC2 only demonstrates good performance in unimodal functions and shows no significant improvement over the original DSF algorithm in the other three types of functions. Although the DSF-ELC algorithm has made considerable improvements based on DSF, it still lags behind the DSF-ELC1 strategy.

Further, when D=30, as can be seen from Table 5, the DSF-ELC algorithm performs exceptionally well on the four types of functions in the CEC 2017 test set, significantly outperforming the other three algorithms. Both in terms of mean error and variance, the DSF-ELC algorithm has seen a substantial improvement compared with its comprehensive ranking when D=10. Although DSF-ELC1 does not match the performance achieved when D=10, it still performs relatively well. Consistent with the test results when D=10, DSF-ELC2 only shows decent performance on single-mode functions.

From the comprehensive Table 4 and Table 5, it can be observed that regardless of whether dealing with low-dimensional or high-dimensional optimization problems, the dynamic population reorganization strategy can significantly enhance the accuracy and stability of the algorithm’s solutions. However, the comprehensive learning strategy does not significantly improve the algorithm’s performance. As the dimension of the optimization problem increases, the difficulty of optimization grows exponentially. Relying solely on the dynamic population reorganization strategy cannot enhance the algorithm’s global search capability. When combined with the comprehensive learning strategy, it increases the algorithm’s exploration ability across the entire domain space. The two strategies work synergistically to effectively improve the overall performance of the algorithm.

### 5.3. Performance Comparison Experiment

In this section, DSF-ELC is compared with some classic and competitive swarm intelligence optimization algorithms, including the Artificial Bee Colony Algorithm (ABC) [7], the Covariance Matrix Adaptation Evolution Strategy (CMAES) [25], the Dynamic Search Fireworks Algorithm (dynFWA) [26], the Loser-Based Fireworks Algorithm (LOTFWA) [27], the Sparrow Search Algorithm (SSA) [28], and the Standard Particle Swarm Optimization 2011 (SPSO2011) [29]. The parameters of these algorithms are set to the values recommended in their original papers. The parameter settings for DSF-ELC are shown in Table 3. All algorithms are tested on the CEC 2017 single-objective optimization benchmark suite [30], including unimodal functions (F1–F3), multimodal functions (F4–F10), hybrid functions (F11–F20), and composite functions (F21–F30), and are tested under the same conditions.

Experiments were conducted on 10-dimensional and 30-dimensional problems of each test function. The results of each experiment were obtained based on 30 independent trial runs. The optimal fitness values obtained by running each algorithm 30 times were averaged, and this value was used as the final value of the algorithm for solving the corresponding test function. The maximum function evaluation times per run (max FE) were 10,000×D, where *D* is the dimension of the optimization problem. When max FE was reached, the algorithm terminated. When the difference between the found optimal solution and the optimal solution of the function was 10E−8 or less, the error was considered to be 0.

Performance comparison experiments include the following: Error mean and variance analysis of DSF-ELC and six other algorithms on the CEC 2017 test set, comparing the performance differences between D=10 and D=30.

Table 6 and Table 7 present the mean and standard deviation of the optimal solutions achieved by DSF-ELC and six other comparative algorithms on 30 functions from the CEC 2017 test set, with the optimal values highlighted in bold. Table 8 and Table 9 rank the test results of the algorithms on each function. Table 10 and Table 11, respectively, provide the average rankings of all algorithms across the four types of functions and on all 30 functions in the CEC 2017. AR (Average Rank) represents the average ranking of the algorithm on the corresponding function, e.g., AR.F1–3 represents the average ranking of the algorithm on functions F1–F3. Best denotes the number of functions where the algorithm performs best, 2nd Best indicates the number of functions where the algorithm performs second best, and Worst represents the number of functions where the algorithm performs worst.

When the dimension of the optimization problem is D=10, the test results are presented in Table 6, Table 8 and Table 10. As shown in Table 10, in terms of the mean error and variance average rank of 30 functions, ABC ranks first with a mean comprehensive rank of 2.53, while LOTFWA ranks second with a mean comprehensive rank of 2.97. DSF-ELC and SPSO2011 perform comparably, ranking third and fourth, respectively. CMAES and dynFWA perform comparably, ranking fifth and sixth, respectively. However, SSA lags far behind other algorithms, ranking seventh.

For single-mode functions (F1–F3), CMAES performs particularly well, with an average rank of 1.00 for both mean and variance, while DSF-ELC performs generally, with an average rank of only 4.67. As shown in Table 8, it did not rank first or second for any function, and only ranked third for F1.

For multimodal functions, hybrid functions, and composite functions, ABC performs optimally, with mean average rankings of 2.57, 2.90, and 1.60, respectively, and ranks first in mean average across 11 functions. Among them, for multimodal functions (F4–F10), DSF-ELC ranks second with a mean average ranking of 2.71, slightly lower than ABC’s 2.57. As shown in Table 8, DSF-ELC ranks first in mean for three functions (i.e., F5, F7, and F8), and also ranks first or second in variance for these three functions.

For mixed functions (F11–F20), DSF-ELC ranks sixth with an average of 4.40, only slightly better than SSA. As shown in Table 8, DSF-ELC ranks second only in the mean of function F16, and ranks second in the mean of functions F11 and F16. There are relatively few functions that rank highly.

For the combined functions (F21–F30), DSF-ELC has an average mean rank of 3.00, which is lower than ABC (average mean rank of 1.60) and LOTFWA (average mean rank of 2.60), ranking third. Meanwhile, DSF-ELC has an average variance rank of 2.60, ranking first, demonstrating good stability. As shown in Table 8, DSF-ELC ranks first in mean for function F27, second in mean for functions F21, F23, and F28, and ranks first in variance for functions F23, F26, F27, and F29, which contributes to an improvement in average rank.

Overall, DSF-ELC performs well in the optimization of multimodal functions (F4–F10), but not so well in the optimization of unimodal, hybrid, and composite functions, especially in the case of hybrid functions, where its average ranking is relatively low. This is because the population dynamic reorganization and comprehensive learning strategies adopted by DSF-ELC are primarily designed to escape local optima in complex optimization problems. When the dimension of the optimization problem is low, these two strategies do not exert their effect. Next, we will conduct tests on a 30-dimensional optimization problem.

### 5.4. Statistical Analysis

In order to conduct a more comprehensive comparison, the Wilcoxon signed rank test was performed on the CEC 2017 30-dimensional test set with a confidence level of 95%. A comprehensive statistical comparison was conducted between the algorithm DSF-ELC and six representative algorithms (ABC, CMAES, dynFWA, LOTFWA, SSA, and SPSO2011). The experimental results are shown in Table 12, and the last row of the table provides the overall results.

In comparison with ABC, dynFWA, and SSA, DSF-ELC achieved statistical victories (“+”) on the vast majority of test functions, especially on multimodal functions, hybrid, and composite functions, with *p*-values generally below the order of 10E−10, demonstrating DSF-ELC’s significant advantages in global exploration ability and convergence accuracy. Compared with CMAES and SPSO2011, DSF-ELC also has an advantage in complex functions. Although its performance is slightly inferior on a few simple or unimodal functions, it still maintains strong competitiveness overall. In comparison with LOTFWA, DSF-ELC has more wins and fewer losses, demonstrating good adaptability to different problem structures.

Overall, DSF-ELC has a significantly higher overall win rate than the comparison algorithms on the CEC 2017 test functions, and the statistical significance is extremely strong. This indicates that DSF-ELC has outstanding potential and practical value in dealing with complex continuous optimization problems and is an efficient optimization algorithm worth promoting.

### 5.5. Stability Analysis

**Figure 4:**
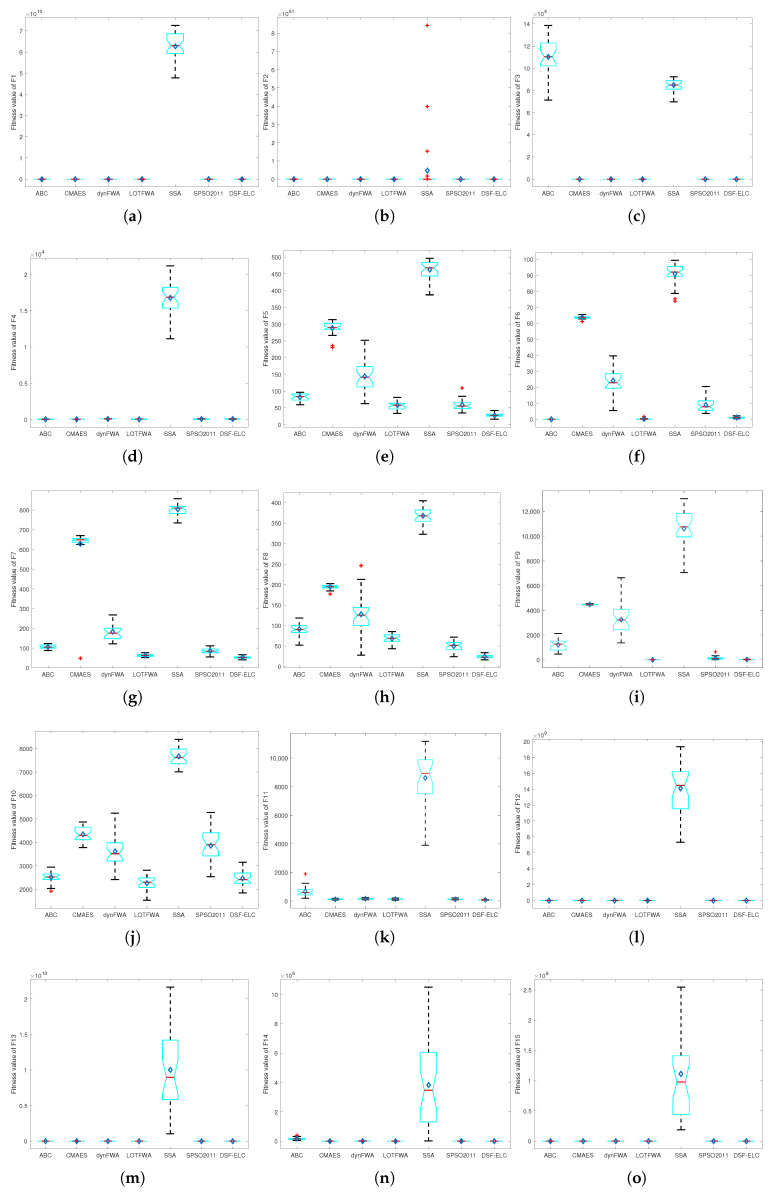
Box plots of errors of the ABC, CMAES, dynFWA, LOTFWA, SSA, SPSO2011, and DSF-ELC on the CEC 2017 test functions (D=30), F1–F15: (**a**) F1. (**b**) F2. (**c**) F3. (**d**) F4. (**e**) F5. (**f**) F6. (**g**) F7. (**h**) F8. (**i**) F9. (**j**) F10. (**k**) F11. (**l**) F12. (**m**) F13. (**n**) F14. (**o**) F15.

**Figure 5:**
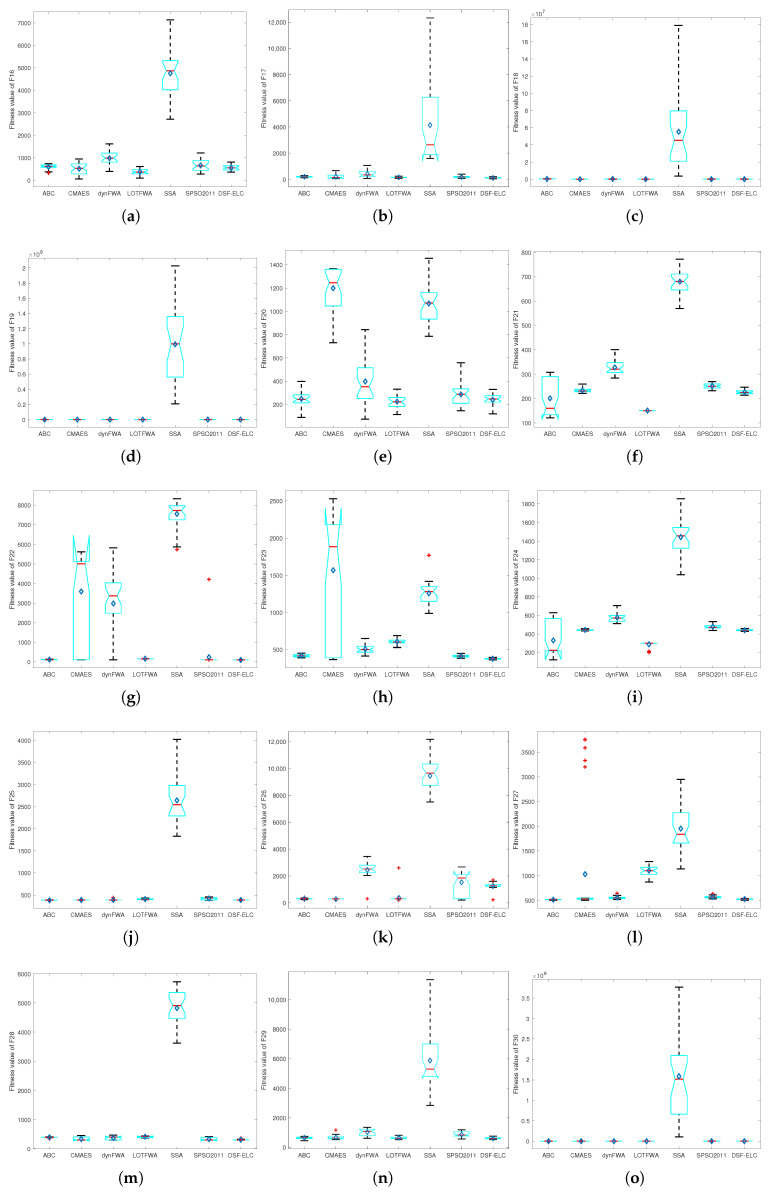
Box plots of errors of the ABC, CMAES, dynFWA, LOTFWA, SSA, SPSO2011, and DSF-ELC on the CEC 2017 test functions (D=30), F16–F30: (**a**) F16. (**b**) F17. (**c**) F18. (**d**) F19. (**e**) F20. (**f**) F21. (**g**) F22. (**h**) F23. (**i**) F24. (**j**) F25. (**k**) F26. (**l**) F27. (**m**) F28. (**n**) F29. (**o**) F30.

Present the statistical information of these algorithms on the CEC 2017 benchmark test set in the form of a box plot to compare stability. Figure 4 and Figure 5 show the test results of a 30 dimensional function.

For all test functions, except for F10, F16, F20, F21, F24, and F26, the fitness error of DSF-ELC is close to 0, while other comparison algorithms show varying degrees of high fitness error, and even SSA reaches the level of 10E9 on high complexity functions. In terms of stability, DSF-ELC is significantly superior to other algorithms, especially for multi-modal complex functions (F11–F30). In contrast, SSA has the worst overall stability and most functions have extremely high fitness errors; ABC, CMAES, and LOTFWA perform relatively well on some low-complexity unimodal functions, but their stability sharply decreases on complex multimodal, mixed, and combined functions; DynFWA and SPSO2011 maintain moderate stability, but there is still a significant gap in convergence accuracy compared with DSF-ELC. From this, it can be seen that DSF-ELC achieved the lowest average fitness error on all 30 test functions of the CEC 2017 test suite for 30D, and its stability was superior to that of other comparative algorithms, demonstrating significant advantages.

### 5.6. Convergence Analysis

To more intuitively reflect the solution quality and convergence of DSF-ELC, this section compares the convergence curves of six metaheuristic algorithms and DSF-ELC. According to the conclusion in Section 5.3, the optimization performance of DSF-ELC on a 30-dimensional problem is significantly improved compared to a 10-dimensional problem. Therefore, this section focuses on the convergence analysis of the 30-dimensional problem.

**Figure 6:**
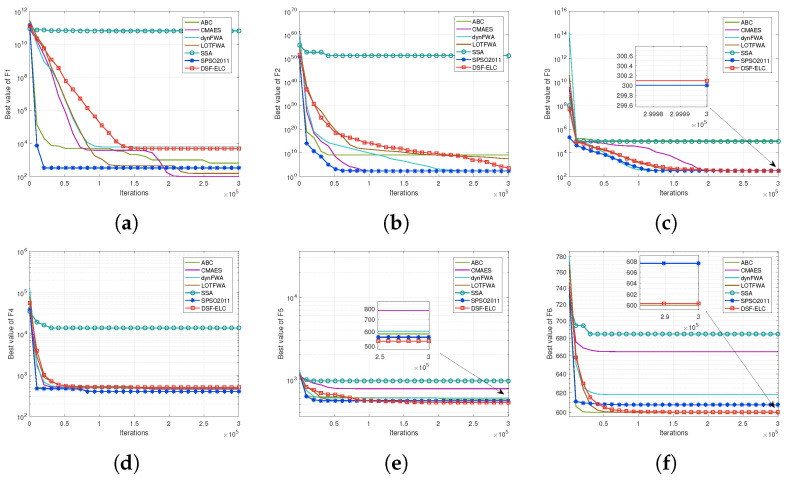

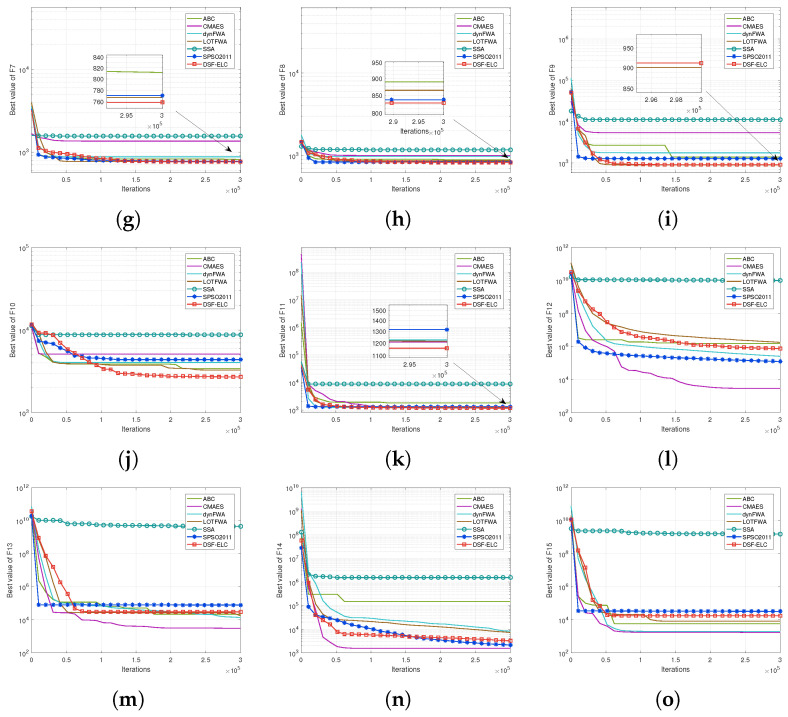
Fitness-iteration comparison among ABC, CMAES, dynFWA, LOTFWA, SSA, SPSO2011, and DSF-ELC for CEC 2017 test functions (D=30), F1–F15: (**a**) F1. (**b**) F2. (**c**) F3. (**d**) F4. (**e**) F5. (**f**) F6. (**g**) F7. (**h**) F8. (**i**) F9. (**j**) F10. (**k**) F11. (**l**) F12. (**m**) F13. (**n**) F14. (**o**) F15.

**Figure 7:**
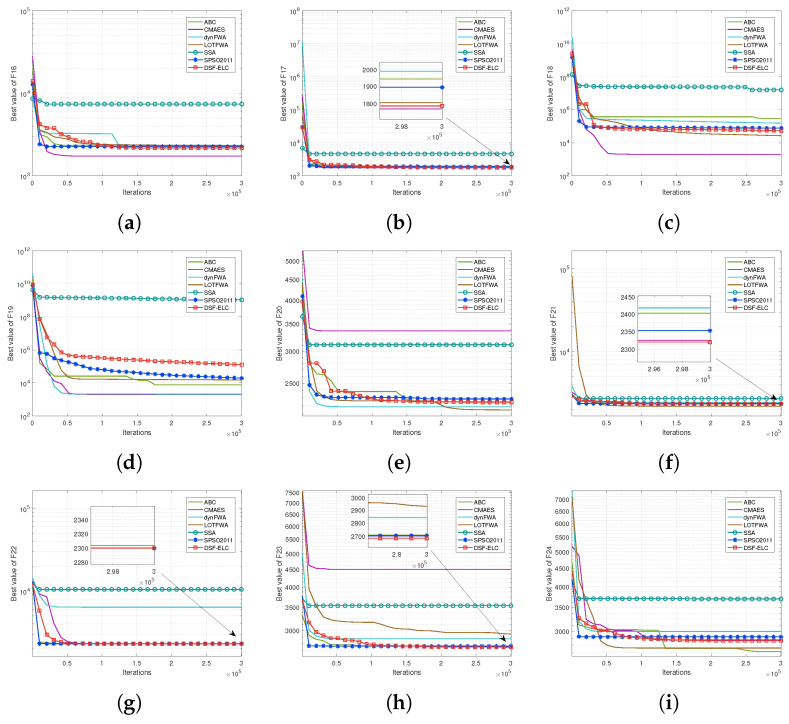

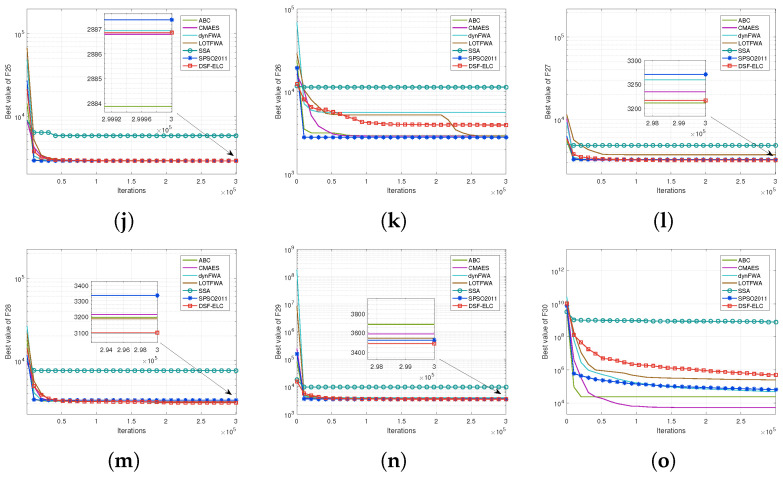
Fitness-iteration comparison among ABC, CMAES, dynFWA, LOTFWA, SSA, SPSO2011, and DSF-ELC CEC 2017 test functions (D=30), F16–F30: (**a**) F16. (**b**) F17. (**c**) F18. (**d**) F19. (**e**) F20. (**f**) F21. (**g**) F22. (**h**) F23. (**i**) F24. (**j**) F25. (**k**) F26. (**l**) F27. (**m**) F28. (**n**) F29. (**o**) F30.

Figure 6 and Figure 7 present the convergence curves of six comparative algorithms and DSF-ELC on 30 functions from the CEC 2017 test set. The horizontal axis represents the number of iterations, and the vertical axis represents the best fitness value to date. It can be observed that DSF-ELC performs optimally on 10 functions, namely F5, F7, F8, F10, F11, F17, F22, F23, F28, and F29. Upon further observation, it is found that during the early stages of search, DSF-ELC extensively explores the entire solution space and converges slowly. However, once it enters the global optimal region, it accelerates the convergence rate, achieving solutions superior to those of other algorithms, especially for functions F5, F7, F8, and F10. In function F10, when the number of iterations is less than 30,000, the obtained optimal solution is comparable to that of SSA. When the number of iterations exceeds 30,000, DSF-ELC accelerates convergence, and by the time the number of iterations reaches 120,000, it has obtained a solution that is far superior to those of other algorithms. Conversely, SSA converges the slowest and falls into local optima during the early stages of search. These conclusions align with the data presented in Table 7, Table 9 and Table 11.

## 6. Conclusions

### 6.1. Summary of Contributions

This paper proposes a dynamic search algorithm based on an energy level collaboration mechanism, namely DSF-ELC. The algorithm implements a population reorganization strategy based on multi-energy levels, allocating sampling particles to different populations according to the ranking of energy level occurrence probabilities. The probability of a subpopulation with lower energy levels increases continuously, gradually acquiring more sampling particles and attracting particles to approach the global optimum region. The method of sampling probability ranking can balance the distribution gradient of particles and avoid excessive differences between subpopulations, which can easily lead to local optima. Based on the implementation of two energy levels, a comprehensive learning strategy for inferior solutions is adopted. Two particles are randomly selected from the subpopulation, and the selected particles are compared with inferior solutions in each dimension of the optimization problem. The optimal one is updated, strengthening the population’s exploration ability and enabling rapid acquisition of the optimal solution. Through the population reorganization strategy and comprehensive learning strategy, the exploration and exploitation capabilities of the algorithm are strengthened further. Experimental results have shown that the DSF-ELC algorithm has relatively strong global search capabilities, with little improvement in performance for single-mode functions, but significant advantages in optimizing complex multimodal optimization functions.

### 6.2. Limitations

Despite the promising results presented in this study, several limitations should be acknowledged.

First, dimensionality scope. The experiments are conducted only on the CEC 2017 benchmark suite at dimensions 10 and 30. Performance on higher dimensions (*D* = 50, 100) has not been evaluated.

Second, benchmark scope. Only the CEC 2017 test suite is used. Validation on other benchmark suites (e.g., CEC 2014, CEC 2020) or real-world engineering problems is not included in the current study.

Third, validation scope. While parameter sensitivity analysis and ablation study are provided to demonstrate the robustness of the algorithm and the contribution of each component, the overall validation scope remains limited to synthetic benchmark functions. Future work will extend the validation to higher dimensions and real-world applications.

## Figures and Tables

**Table 1:** Average rankings of DSF-ELC algorithm under different subpopulation numbers *N* and sorting adjustment factor β when α=10 (D=10).

Fun.	N = 30 β=2		N = 30 β=3		N = 30 β=4		N = 50 β=2		N = 50 β=3		N = 50 β=4		N = 100 β=2		N = 100 β=3		N = 100 β=4	
	Mean	Std	Mean	Std	Mean	Std	Mean	Std	Mean	Std	Mean	Std	Mean	Std	Mean	Std	Mean	Std
AR.F1– 3	**1.67**	2.00	2.33	2.33	2.00	**1.67**	4.67	4.67	5.00	5.00	5.33	5.33	8.00	8.67	8.33	7.67	7.67	7.67
AR.F4–10	2.29	3.14	2.29	**2.00**	**1.57**	2.71	4.86	4.57	4.57	5.14	5.43	5.00	7.43	6.86	8.00	8.00	8.57	7.57
AR.F11–20	6.10	5.90	4.40	5.00	**4.20**	6.20	5.80	5.80	4.90	5.20	**4.20**	**3.10**	5.70	5.20	5.50	3.80	**4.20**	4.80
AR.F21–30	4.00	**3.60**	3.70	4.70	4.30	4.40	5.00	5.20	4.70	5.00	**3.20**	6.00	6.80	5.80	6.50	5.60	6.80	4.70
AR.F1–30	4.07	4.10	3.47	**3.93**	**3.40**	4.33	5.20	5.20	4.77	5.10	4.27	4.73	6.70	6.13	6.70	5.77	6.43	5.70
Best	5	8	6	5	8	6	1	0	2	1	3	3	0	1	2	5	3	1
2nd Best	4	4	9	9	7	2	1	3	1	2	2	2	2	2	2	2	2	4
Worst	3	2	2	2	2	4	1	2	1	3	0	1	5	7	7	6	9	3

The bold mark indicates that they are the best results among the algorithms.

**Table 2:** Performance comparison of DSF-ELC algorithm under different scaling factors α when N=30, β=4 (D=10).

Fun.	α=0.1		α=1		α=10	
	Mean	Std	Mean	Std	Mean	Std
AR.F1–3	2.33	2.33	2.00	2.00	**1.67**	**1.67**
AR.F4–10	2.57	2.43	**1.43**	**1.57**	2.00	2.00
AR.F11–20	**1.70**	**1.90**	2.20	**1.90**	2.10	2.20
AR.F21–30	2.00	2.10	2.30	2.10	**1.70**	**1.80**
AR.F1–30	1.97	2.00	1.87	**1.77**	**1.77**	1.83
Best	11	11	7	9	12	10
2nd Best	6	4	15	15	9	11
Worst	13	15	8	6	9	9

The bold mark indicates that they are the best results among the algorithms.

**Table 3:** Parameter settings for compared algorithms.

Algorithm	Parameter Settings
DSF	N=30
DSF-ELC1	N=30,β=4
DSF-ELC2	N=30,α=10
DSF-ELC	N=30,β=4,α=10

**Table 4:** Comparison of average performance of four algorithms (D=10).

Fun.	DSF		DSF-ELC1		DSF-ELC2		DSF-ELC	
	Mean	Std	Mean	Std	Mean	Std	Mean	Std
F1	9.94 × 10^1^	1.30 × 10^2^	1.98 × 10^2^	3.04 × 10^2^	**8.87 × 10^1^**	**1.19 × 10^2^**	5.25 × 10^2^	7.62 × 10^2^
F2	1.93 × 10^1^	1.54 × 10^1^	5.31 × 10^1^	2.60 × 10^1^	1.88 × 10^1^	**1.49 × 10^1^**	**1.01 × 10^1^**	1.83 × 10^1^
F3	**0.00**	**0.00**	2.26 × 10^−3^	1.63 × 10^−3^	**0.00**	**0.00**	**0.00**	**0.00**
F4	3.90	3.89 × 10^−1^	3.76	6.61 × 10^−1^	3.99	**2.98 × 10^−1^**	**1.35**	4.04 × 10^−1^
F5	2.79 × 10^1^	6.86	**3.96**	**1.32**	2.63 × 10^1^	8.33	4.96	2.12
F6	7.19 × 10^−1^	5.14 × 10^−1^	1.95 × 10^−1^	2.01 × 10^−1^	1.00	6.11 × 10^−1^	**8.74 × 10^−2^**	**1.68 × 10^−1^**
F7	4.12 × 10^1^	4.86	**1.32 × 10^1^**	**1.42**	3.95 × 10^1^	5.55	1.44 × 10^1^	2.09
F8	2.83 × 10^1^	9.72	**3.68**	3.36	2.93 × 10^1^	9.96	5.12	**2.33**
F9	**0.00**	**0.00**	1.36 × 10^−5^	9.94 × 10^−6^	**0.00**	**0.00**	**0.00**	**0.00**
F10	1.36 × 10^3^	1.56 × 10^2^	**3.40 × 10^2^**	**1.51 × 10^2^**	1.35 × 10^3^	1.77 × 10^2^	4.47 × 10^2^	1.94 × 10^2^
F11	2.48 × 10^1^	8.35	**1.10 × 10^1^**	**4.84**	2.23 × 10^1^	7.42	1.62 × 10^1^	8.17
F12	3.54 × 10^5^	2.22 × 10^5^	1.13 × 10^5^	9.44 × 10^4^	3.41 × 10^5^	1.73 × 10^5^	**5.23 × 10^4^**	**5.67 × 10^4^**
F13	7.10 × 10^3^	**1.46 × 10^3^**	**5.92 × 10^3^**	2.36 × 10^3^	8.03 × 10^3^	2.56 × 10^3^	7.79 × 10^3^	4.65 × 10^3^
F14	9.64 × 10^2^	9.08 × 10^2^	**1.06 × 10^2^**	**7.11 × 10^1^**	7.05 × 10^2^	6.48 × 10^2^	1.10 × 10^2^	7.76 × 10^1^
F15	4.91 × 10^3^	3.34 × 10^3^	1.12 × 10^3^	6.05 × 10^2^	5.15 × 10^3^	3.49 × 10^3^	**7.88 × 10^2^**	**4.29 × 10^2^**
F16	9.87 × 10^1^	3.22 × 10^1^	**9.94**	**5.58**	1.24 × 10^2^	5.02 × 10^1^	2.83 × 10^1^	4.54 × 10^1^
F17	8.18 × 10^1^	1.41 × 10^1^	**2.88 × 10^1^**	**8.56**	8.61 × 10^1^	1.34 × 10^1^	3.24 × 10^1^	1.13 × 10^1^
F18	5.52 × 10^3^	2.31 × 10^3^	**2.99 × 10^3^**	**2.10 × 10^3^**	5.70 × 10^3^	2.80 × 10^3^	5.74 × 10^3^	4.79 × 10^3^
F19	2.59 × 10^3^	1.80 × 10^3^	3.57 × 10^2^	5.96 × 10^2^	2.53 × 10^3^	1.67 × 10^3^	**2.03 × 10^2^**	**2.84 × 10^2^**
F20	9.23 × 10^1^	1.46 × 10^1^	**3.18 × 10^1^**	**6.29**	9.17 × 10^1^	1.70 × 10^1^	3.45 × 10^1^	1.54 × 10^1^
F21	1.33 × 10^2^	4.38 × 10^1^	1.01 × 10^2^	**5.43 × 10^−1^**	1.19 × 10^2^	2.31 × 10^1^	**1.00 × 10^2^**	7.85 × 10^−1^
F22	1.03 × 10^2^	**6.49**	**6.39 × 10^1^**	4.41 × 10^1^	1.03 × 10^2^	8.28	8.59 × 10^1^	3.01 × 10^1^
F23	3.33 × 10^2^	5.10	**3.06 × 10^2^**	**2.41**	3.29 × 10^2^	8.49	3.07 × 10^2^	2.85
F24	2.97 × 10^2^	7.01 × 10^1^	**1.89 × 10^2^**	1.08 × 10^2^	3.11 × 10^2^	**6.92 × 10^1^**	2.10 × 10^2^	1.27 × 10^2^
F25	4.21 × 10^2^	2.22 × 10^1^	**4.09 × 10^2^**	**1.96 × 10^1^**	4.17 × 10^2^	2.20 × 10^1^	4.18 × 10^2^	2.31 × 10^1^
F26	3.00 × 10^2^	5.51 × 10^−7^	**2.90 × 10^2^**	5.24 × 10^1^	3.00 × 10^2^	4.16 × 10^−7^	3.00 × 10^2^	**2.52 × 10^−12^**
F27	3.96 × 10^2^	1.48	**3.94 × 10^2^**	1.41	3.96 × 10^2^	**1.34**	3.95 × 10^2^	1.77
F28	3.00 × 10^2^	5.20 × 10^−1^	**2.97 × 10^2^**	3.86 × 10^1^	3.05 × 10^2^	2.47 × 10^1^	3.00 × 10^2^	**5.02 × 10^−1^**
F29	3.27 × 10^2^	2.32 × 10^1^	**2.54 × 10^2^**	8.97	3.22 × 10^2^	1.68 × 10^1^	2.57 × 10^2^	**8.92**
F30	2.34 × 10^4^	6.53 × 10^3^	**6.29 × 10^3^**	5.23 × 10^3^	1.82 × 10^5^	4.20 × 10^5^	8.68 × 10^3^	**3.45 × 10^3^**
AR.F1–3	2.00	1.67	3.67	3.67	**1.33**	**1.00**	2.00	2.67
AR.F4–10	3.14	2.43	1.71	2.14	3.14	3.00	**1.57**	**2.00**
AR.F11–20	3.30	3.00	**1.30**	**1.40**	3.40	3.20	2.00	2.40
AR.F21–30	3.60	2.80	**1.10**	2.40	3.30	2.50	2.00	**2.30**
AR.F1–30	3.00	2.43	**1.50**	**2.00**	2.87	2.47	1.77	2.23
Best/ 2nd Best/ Worst	2/3/14	4/9/7	20/6/3	12/9/7	3/2/11	7/4/9	9/17/2	11/6/7

The bold mark indicates that they are the best results among the algorithms.

**Table 5:** Comparison of average performance of four algorithms (D=30).

Fun.	DSF		DSF-ELC1		DSF-ELC2		DSF-ELC	
	Mean	Std	Mean	Std	Mean	Std	Mean	Std
F1	1.78 × 10^3^	9.25 × 10^2^	2.00 × 10^3^	9.99 × 10^2^	**1.58 × 10^3^**	**6.41 × 10^2^**	1.76 × 10^3^	8.79 × 10^2^
F2	3.61 × 10^−5^	3.85 × 10^−5^	2.55 × 10^5^	5.46 × 10^5^	**2.74 × 10^−5^**	**3.41 × 10^−5^**	2.44 × 10^5^	1.06 × 10^6^
F3	2.28 × 10^−1^	2.16 × 10^−1^	1.40 × 10^−1^	7.67 × 10^−2^	2.29 × 10^−1^	2.91 × 10^−1^	**1.11 × 10^−1^**	**6.32 × 10^−2^**
F4	9.00 × 10^1^	3.26 × 10^1^	8.30 × 10^1^	3.69 × 10^1^	9.94 × 10^1^	**2.47 × 10^1^**	**8.12 × 10^1^**	3.99 × 10^1^
F5	1.61 × 10^2^	6.24 × 10^1^	2.86 × 10^1^	**5.64**	1.89 × 10^2^	1.70 × 10^1^	**2.79 × 10^1^**	5.97
F6	3.27	2.10	1.18	5.55 × 10^−1^	3.60	2.21	**1.03**	**4.94 × 10^−1^**
F7	2.07 × 10^2^	3.87 × 10^1^	5.67 × 10^1^	7.61	2.10 × 10^2^	3.41 × 10^1^	**5.29 × 10^1^**	**5.90**
F8	1.77 × 10^2^	5.40 × 10^1^	2.49 × 10^1^	6.69	1.74 × 10^2^	4.11 × 10^1^	**2.45 × 10^1^**	**4.49**
F9	4.09 × 10^1^	2.87 × 10^1^	1.65 × 10^1^	2.71 × 10^1^	3.78 × 10^1^	3.55 × 10^1^	**1.42 × 10^1^**	**1.43 × 10^1^**
F10	7.16 × 10^3^	**2.51 × 10^2^**	2.50 × 10^3^	3.75 × 10^2^	7.11 × 10^3^	2.67 × 10^2^	**2.47 × 10^3^**	3.16 × 10^2^
F11	9.11 × 10^1^	2.01 × 10^1^	**7.20 × 10^1^**	**1.48 × 10^1^**	1.06 × 10^2^	3.06 × 10^1^	7.38 × 10^1^	1.71 × 10^1^
F12	1.19 × 10^6^	4.25 × 10^5^	7.16 × 10^5^	3.33 × 10^5^	1.21 × 10^6^	**2.86 × 10^5^**	**7.11 × 10^5^**	2.99 × 10^5^
F13	**2.48 × 10^4^**	5.81 × 10^3^	2.65 × 10^4^	5.45 × 10^3^	2.74 × 10^4^	**5.41 × 10^3^**	2.64 × 10^4^	7.30 × 10^3^
F14	3.19 × 10^3^	3.57 × 10^3^	**7.50 × 10^2^**	**6.25 × 10^2^**	2.70 × 10^3^	2.24 × 10^3^	8.93 × 10^2^	7.10 × 10^2^
F15	**1.33 × 10^4^**	3.02 × 10^3^	1.43 × 10^4^	**3.00 × 10^3^**	1.43 × 10^4^	4.04 × 10^3^	1.41 × 10^4^	3.69 × 10^3^
F16	1.72 × 10^3^	3.07 × 10^2^	**5.32 × 10^2^**	1.79 × 10^2^	1.80 × 10^3^	2.10 × 10^2^	5.57 × 10^2^	**1.27 × 10^2^**
F17	7.00 × 10^2^	1.35 × 10^2^	1.19 × 10^2^	5.10 × 10^1^	6.81 × 10^2^	1.31 × 10^2^	**1.16 × 10^2^**	**4.47 × 10^1^**
F18	9.54 × 10^4^	2.91 × 10^4^	**6.10 × 10^4^**	**2.38 × 10^4^**	9.64 × 10^4^	4.34 × 10^4^	6.63 × 10^4^	2.59 × 10^4^
F19	1.72 × 10^5^	4.18 × 10^4^	1.09 × 10^5^	3.46 × 10^4^	1.63 × 10^5^	6.04 × 10^4^	**1.08 × 10^5^**	**3.64 × 10^4^**
F20	7.08 × 10^2^	9.02 × 10^1^	2.63 × 10^2^	5.67 × 10^1^	6.86 × 10^2^	8.45 × 10^1^	**2.38 × 10^2^**	**5.45 × 10^1^**
F21	3.78 × 10^2^	3.50 × 10^1^	**2.26 × 10^2^**	**6.19**	3.61 × 10^2^	5.59 × 10^1^	2.26 × 10^2^	7.77
F22	**1.00 × 10^2^**	**3.44 × 10^−6^**	1.00 × 10^2^	4.48 × 10^−1^	1.00 × 10^2^	4.48 × 10^−1^	**1.00 × 10^2^**	1.01 × 10^−5^
F23	5.18 × 10^2^	4.93 × 10^1^	3.82 × 10^2^	1.18 × 10^1^	5.01 × 10^2^	6.29 × 10^1^	**3.78 × 10^2^**	**7.74**
F24	5.70 × 10^2^	6.41 × 10^1^	**4.41 × 10^2^**	**6.34**	5.59 × 10^2^	6.98 × 10^1^	4.41 × 10^2^	7.76
F25	4.23 × 10^2^	2.13 × 10^1^	**3.87 × 10^2^**	**7.02 × 10^−1^**	4.27 × 10^2^	1.94 × 10^1^	3.87 × 10^2^	2.72
F26	2.22 × 10^3^	6.27 × 10^2^	1.32 × 10^3^	**2.52 × 10^2^**	2.29 × 10^3^	6.51 × 10^2^	**1.25 × 10^3^**	3.02 × 10^2^
F27	5.48 × 10^2^	1.36 × 10^1^	5.24 × 10^2^	9.04	5.48 × 10^2^	1.25 × 10^1^	**5.23 × 10^2^**	**8.67**
F28	3.52 × 10^2^	4.49 × 10^1^	3.25 × 10^2^	3.15 × 10^1^	3.55 × 10^2^	4.17 × 10^1^	**3.11 × 10^2^**	**1.50 × 10^1^**
F29	1.20 × 10^3^	1.99 × 10^2^	6.16 × 10^2^	5.62 × 10^1^	1.21 × 10^3^	2.03 × 10^2^	**6.08 × 10^2^**	**5.23 × 10^1^**
F30	1.00 × 10^6^	3.41 × 10^5^	5.50 × 10^5^	2.54 × 10^5^	9.47 × 10^5^	3.82 × 10^5^	**5.11 × 10^5^**	**1.54 × 10^5^**
AR.F1–3	2.67	2.67	3.33	3.00	**2.00**	**2.00**	**2.00**	2.33
AR.F4–10	3.43	3.00	2.00	2.29	3.57	2.86	**1.00**	**1.86**
AR.F11–20	3.00	3.40	1.80	**1.60**	3.60	3.00	**1.60**	2.00
AR.F21–30	3.40	3.10	1.90	1.80	3.40	3.60	**1.30**	**1.50**
AR.F1–30	2.97	2.93	1.90	1.83	3.13	2.80	**1.33**	**1.77**
Best/ 2nd Best/ Worst	3/1/12	2/3/11	7/18/3	9/14/3	2/0/15	5/1/13	19/10/0	14/12/3

The bold mark indicates that they are the best results among the algorithms.

**Table 6:** Comparison of average performance between DSF-ELC and six famous optimization algorithms (D=10).

Fun.	ABC		CMAES		dynFWA		LOTFWA		SSA		SPSO2011		DSF-ELC	
	Mean	Std	Mean	Std	Mean	Std	Mean	Std	Mean	Std	Mean	Std	Mean	Std
F1	2.52 × 10^2^	3.48 × 10^2^	**0.00**	**0.00**	2.07 × 10^3^	2.26 × 10^3^	6.89 × 10^1^	1.08 × 10^2^	1.28 × 10^10^	3.89 × 10^9^	1.24 × 10^3^	2.17 × 10^3^	1.81 × 10^2^	2.01 × 10^2^
F2	2.57 × 10^−4^	3.05 × 10^−4^	**0.00**	**0.00**	1.03 × 10^1^	2.05 × 10^1^	4.62	9.52	2.38 × 10^12^	6.51 × 10^12^	3.07 × 10^−5^	2.46 × 10^−5^	6.09 × 10^1^	2.94 × 10^1^
F3	6.62 × 10^3^	3.51 × 10^3^	**0.00**	**0.00**	**0.00**	**0.00**	1.05 × 10^−5^	1.17 × 10^−5^	1.07 × 10^4^	2.93 × 10^3^	**0.00**	**0.00**	2.84 × 10^−3^	2.47 × 10^−3^
F4	7.06 × 10^−1^	1.04	**0.00**	**0.00**	2.70	9.66 × 10^−1^	3.05	8.81	9.41 × 10^2^	5.84 × 10^2^	4.28 × 10^−3^	1.57 × 10^−3^	3.89	7.99 × 10^−1^
F5	7.93	**1.68**	1.03 × 10^2^	4.50 × 10^1^	1.63 × 10^1^	6.24	9.68	3.26	9.72 × 10^1^	1.58 × 10^1^	5.94	2.36	**4.00**	1.69
F6	**0.00**	**0.00**	5.79 × 10^1^	3.22	1.28 × 10^−1^	3.91 × 10^−1^	5.14 × 10^−5^	3.52 × 10^−5^	5.06 × 10^1^	9.30	3.28 × 10^−1^	4.20 × 10^−1^	1.76 × 10^−1^	1.62 × 10^−1^
F7	1.79 × 10^1^	**2.16**	1.04 × 10^2^	2.42 × 10^1^	2.92 × 10^1^	1.00 × 10^1^	1.79 × 10^1^	3.58	1.30 × 10^2^	1.56 × 10^1^	1.52 × 10^1^	3.39	**1.35 × 10^1^**	2.20
F8	8.12	2.36	3.25 × 10^1^	3.34	2.02 × 10^1^	7.39	9.17	2.87	5.90 × 10^1^	8.95	6.91	3.29	**3.01**	**1.74**
F9	3.17 × 10^−2^	7.70 × 10^−2^	8.48 × 10^2^	2.36 × 10^1^	**2.01 × 10^−10^**	**9.91 × 10^−10^**	1.86 × 10^−8^	1.98 × 10^−8^	7.40 × 10^2^	1.94 × 10^2^	5.12 × 10^−2^	1.30 × 10^−1^	1.95 × 10^−5^	2.74 × 10^−5^
F10	**2.45 × 10^2^**	**9.41 × 10^1^**	1.66 × 10^3^	5.23 × 10^2^	5.77 × 10^2^	2.32 × 10^2^	3.06 × 10^2^	1.39 × 10^2^	1.54 × 10^3^	1.46 × 10^2^	4.25 × 10^2^	2.79 × 10^2^	3.54 × 10^2^	1.91 × 10^2^
F11	**5.41**	**3.51**	2.36 × 10^1^	1.99 × 10^1^	1.46 × 10^1^	1.04 × 10^1^	6.96	4.23	1.22 × 10^3^	1.47 × 10^3^	2.73 × 10^1^	1.53 × 10^1^	1.18 × 10^1^	3.96
F12	3.15 × 10^4^	1.85 × 10^4^	**4.80 × 10^2^**	**2.09 × 10^2^**	2.18 × 10^4^	1.79 × 10^4^	3.08 × 10^4^	4.18 × 10^4^	3.40 × 10^8^	3.19 × 10^8^	1.22 × 10^4^	1.16 × 10^4^	1.38 × 10^5^	1.31 × 10^5^
F13	1.34 × 10^3^	1.57 × 10^3^	**3.46 × 10^1^**	**4.22 × 10^1^**	8.53 × 10^3^	8.06 × 10^3^	1.63 × 10^3^	9.73 × 10^2^	1.03 × 10^6^	1.89 × 10^6^	3.94 × 10^3^	2.87 × 10^3^	6.31 × 10^3^	2.78 × 10^3^
F14	3.10 × 10^2^	3.68 × 10^2^	4.73 × 10^1^	**1.68 × 10^1^**	**2.94 × 10^1^**	2.72 × 10^1^	9.38 × 10^1^	2.89 × 10^1^	9.83 × 10^1^	2.73 × 10^1^	6.79 × 10^1^	2.29 × 10^1^	1.05 × 10^2^	7.29 × 10^1^
F15	8.74 × 10^1^	6.07 × 10^1^	**4.00 × 10^1^**	**2.70 × 10^1^**	4.10 × 10^1^	1.16 × 10^2^	1.40 × 10^2^	5.69 × 10^1^	1.79 × 10^3^	1.66 × 10^3^	2.40 × 10^2^	2.00 × 10^2^	1.21 × 10^3^	8.47 × 10^2^
F16	1.71 × 10^1^	3.17 × 10^1^	4.70 × 10^2^	1.24 × 10^2^	1.09 × 10^2^	8.37 × 10^1^	**7.67**	**5.18**	4.74 × 10^2^	1.05 × 10^2^	2.03 × 10^1^	3.92 × 10^1^	1.07 × 10^1^	6.04
F17	**3.22**	**1.66**	1.95 × 10^2^	1.14 × 10^2^	3.50 × 10^1^	3.28 × 10^1^	3.02 × 10^1^	5.99	1.32 × 10^2^	4.66 × 10^1^	4.03 × 10^1^	9.96	3.26 × 10^1^	6.47
F18	1.15 × 10^3^	7.08 × 10^2^	**6.50 × 10^1^**	**4.58 × 10^1^**	6.47 × 10^3^	6.48 × 10^3^	1.34 × 10^3^	9.82 × 10^2^	2.90 × 10^5^	8.82 × 10^5^	2.93 × 10^3^	3.90 × 10^3^	2.70 × 10^3^	1.33 × 10^3^
F19	1.05 × 10^2^	1.40 × 10^2^	**1.64 × 10^1^**	**1.67 × 10^1^**	1.59 × 10^2^	3.90 × 10^2^	6.27 × 10^1^	3.03 × 10^1^	2.32 × 10^4^	6.21 × 10^4^	6.77 × 10^1^	1.39 × 10^2^	2.01 × 10^2^	2.15 × 10^2^
F20	**5.21 × 10^−1^**	**5.11 × 10^−1^**	4.28 × 10^2^	4.33 × 10^1^	1.70 × 10^1^	1.17 × 10^1^	3.55 × 10^1^	8.67	2.12 × 10^2^	5.23 × 10^1^	3.51 × 10^1^	6.34	3.19 × 10^1^	7.33
F21	1.06 × 10^2^	1.83 × 10^1^	2.07 × 10^2^	2.48	1.78 × 10^2^	5.97 × 10^1^	**1.00 × 10^2^**	**8.29 × 10^−3^**	2.76 × 10^2^	3.38 × 10^1^	1.96 × 10^2^	3.28 × 10^1^	1.01 × 10^2^	5.49 × 10^−1^
F22	5.94 × 10^1^	2.88 × 10^1^	1.00 × 10^2^	**7.27 × 10^−2^**	1.12 × 10^2^	9.09 × 10^1^	**3.38 × 10^1^**	2.91 × 10^1^	1.01 × 10^3^	4.15 × 10^2^	9.81 × 10^1^	1.43 × 10^1^	7.55 × 10^1^	3.77 × 10^1^
F23	**2.63 × 10^2^**	1.14 × 10^2^	5.90 × 10^2^	2.87 × 10^2^	3.20 × 10^2^	8.69	3.27 × 10^2^	7.82 × 10^1^	4.19 × 10^2^	2.32 × 10^1^	3.08 × 10^2^	4.04	3.07 × 10^2^	**3.46**
F24	**9.49 × 10^1^**	**1.52 × 10^1^**	1.08 × 10^2^	4.25 × 10^1^	2.98 × 10^2^	1.11 × 10^2^	1.07 × 10^2^	5.20 × 10^1^	5.02 × 10^2^	7.28 × 10^1^	2.95 × 10^2^	8.87 × 10^1^	1.99 × 10^2^	1.16 × 10^2^
F25	**2.36 × 10^2^**	1.07 × 10^2^	4.41 × 10^2^	1.17 × 10^1^	4.22 × 10^2^	2.45 × 10^1^	3.95 × 10^2^	**1.05**	1.03 × 10^3^	2.55 × 10^2^	4.27 × 10^2^	2.36 × 10^1^	4.07 × 10^2^	1.84 × 10^1^
F26	**1.10 × 10^2^**	9.42 × 10^1^	2.86 × 10^2^	4.28 × 10^1^	2.75 × 10^2^	8.32 × 10^1^	1.67 × 10^2^	1.49 × 10^2^	1.60 × 10^3^	4.22 × 10^2^	2.98 × 10^2^	2.05 × 10^1^	3.00 × 10^2^	**7.86 × 10^−4^**
F27	3.94 × 10^2^	2.42	4.01 × 10^2^	1.76 × 10^1^	4.00 × 10^2^	1.76 × 10^1^	4.13 × 10^2^	3.30 × 10^1^	5.74 × 10^2^	6.41 × 10^1^	3.96 × 10^2^	3.49	**3.94 × 10^2^**	**1.54**
F28	**2.86 × 10^2^**	7.70 × 10^1^	5.63 × 10^2^	9.09 × 10^1^	3.97 × 10^2^	1.57 × 10^2^	3.00 × 10^2^	**4.23 × 10^−4^**	8.77 × 10^2^	1.63 × 10^2^	4.75 × 10^2^	1.50 × 10^2^	2.98 × 10^2^	3.37 × 10^1^
F29	**2.40 × 10^2^**	3.61 × 10^1^	2.78 × 10^2^	2.83 × 10^1^	3.08 × 10^2^	6.11 × 10^1^	2.52 × 10^2^	6.91	5.71 × 10^2^	1.08 × 10^2^	2.66 × 10^2^	1.21 × 10^1^	2.54 × 10^2^	**6.87**
F30	6.60 × 10^3^	4.22 × 10^3^	**4.41 × 10^2^**	**4.10 × 10^1^**	2.18 × 10^5^	4.85 × 10^5^	1.24 × 10^3^	5.97 × 10^2^	7.91 × 10^6^	6.21 × 10^6^	4.84 × 10^5^	6.31 × 10^5^	6.70 × 10^3^	3.63 × 10^3^

The bold mark indicates that they are the best results among the algorithms.

**Table 7:** Comparison of average performance between DSF-ELC and six famous optimization algorithms (D=30).

Fun.	ABC		CMAES		dynFWA		LOTFWA		SSA		SPSO2011		DSF-ELC	
	Mean	Std	Mean	Std	Mean	Std	Mean	Std	Mean	Std	Mean	Std	Mean	Std
F1	3.06 × 10^2^	2.77 × 10^2^	**0.00**	**0.00**	2.55 × 10^3^	3.21 × 10^3^	1.68 × 10^2^	2.61 × 10^2^	6.26 × 10^10^	6.69 × 10^9^	2.73 × 10^3^	2.72 × 10^3^	1.76 × 10^3^	8.79 × 10^2^
F2	4.23 × 10^9^	1.55 × 10^10^	**0.00**	**0.00**	1.36 × 10^2^	3.95 × 10^2^	1.42 × 10^7^	6.08 × 10^7^	4.72 × 10^50^	1.69 × 10^51^	7.43 × 10^−5^	4.24 × 10^−5^	2.44 × 10^5^	1.06 × 10^6^
F3	1.10 × 10^5^	1.41 × 10^4^	**0.00**	**0.00**	6.23 × 10^−12^	1.33 × 10^−11^	5.52 × 10^−4^	7.43 × 10^−4^	8.50 × 10^4^	5.01 × 10^3^	**0.00**	**0.00**	1.11 × 10^−1^	6.32 × 10^−2^
F3	1.10 × 10^5^	1.41 × 10^4^	**0.00**	**0.00**	**0.00**	**0.00**	5.52 × 10^−4^	7.43 × 10^−4^	8.50 × 10^4^	5.01 × 10^3^	**0.00**	**0.00**	1.11 × 10^−1^	6.32 × 10^−2^
F4	**2.90 × 10^1^**	2.83 × 10^1^	4.83 × 10^1^	2.47 × 10^1^	8.11 × 10^1^	**2.35 × 10^1^**	4.86 × 10^1^	3.12 × 10^1^	1.68 × 10^4^	2.33 × 10^3^	5.32 × 10^1^	4.79 × 10^1^	8.12 × 10^1^	3.99 × 10^1^
F5	8.19 × 10^1^	1.12 × 10^1^	2.88 × 10^2^	1.82 × 10^1^	1.44 × 10^2^	5.05 × 10^1^	5.79 × 10^1^	1.30 × 10^1^	4.63 × 10^2^	2.71 × 10^1^	5.92 × 10^1^	1.61 × 10^1^	**2.79 × 10^1^**	**5.97**
F6	**0.00**	**0.00**	6.37 × 10^1^	8.16 × 10^−1^	2.42 × 10^1^	7.06	1.60 × 10^−1^	3.62 × 10^−1^	9.09 × 10^1^	6.34	9.03	4.41	1.03	4.94 × 10^−1^
F7	1.06 × 10^2^	1.03 × 10^1^	6.29 × 10^2^	1.10 × 10^2^	1.83 × 10^2^	3.81 × 10^1^	6.35 × 10^1^	6.63	8.03 × 10^2^	3.10 × 10^1^	8.57 × 10^1^	1.43 × 10^1^	**5.29 × 10^1^**	**5.90**
F8	9.13 × 10^1^	1.24 × 10^1^	1.95 × 10^2^	6.78	1.28 × 10^2^	4.53 × 10^1^	6.84 × 10^1^	1.16 × 10^1^	3.69 × 10^2^	1.94 × 10^1^	5.02 × 10^1^	1.20 × 10^1^	**2.45 × 10^1^**	**4.49**
F9	1.20 × 10^3^	4.52 × 10^2^	4.48 × 10^3^	3.79 × 10^1^	3.25 × 10^3^	1.26 × 10^3^	**2.41 × 10^−2^**	**1.01 × 10^−1^**	1.06 × 10^4^	1.51 × 10^3^	1.31 × 10^2^	1.31 × 10^2^	1.42 × 10^1^	1.43 × 10^1^
F10	2.52 × 10^3^	**2.44 × 10^2^**	4.35 × 10^3^	2.82 × 10^2^	3.62 × 10^3^	6.37 × 10^2^	3.25 × 10^3^	3.86 × 10^2^	7.67 × 10^3^	3.73 × 10^2^	3.86 × 10^3^	7.49 × 10^2^	**2.47 × 10^3^**	3.16 × 10^2^
F11	6.48 × 10^2^	3.40 × 10^2^	1.19 × 10^2^	3.69 × 10^1^	1.44 × 10^2^	4.43 × 10^1^	1.28 × 10^2^	3.85 × 10^1^	8.63 × 10^3^	1.85 × 10^3^	1.14 × 10^2^	3.78 × 10^1^	**7.38 × 10^1^**	**1.71 × 10^1^**
F12	9.18 × 10^5^	4.08 × 10^5^	**1.45 × 10^3^**	**5.23 × 10^2^**	8.17 × 10^5^	5.38 × 10^5^	4.24 × 10^6^	3.12 × 10^6^	1.41 × 10^10^	3.26 × 10^9^	3.15 × 10^5^	2.64 × 10^5^	7.11 × 10^5^	2.99 × 10^5^
F13	1.72 × 10^4^	8.67 × 10^3^	**1.69 × 10^3^**	**9.31 × 10^2^**	2.99 × 10^4^	2.05 × 10^4^	1.28 × 10^4^	4.41 × 10^3^	1.00 × 10^10^	5.25 × 10^9^	5.68 × 10^4^	2.07 × 10^4^	2.64 × 10^4^	7.30 × 10^3^
F14	1.76 × 10^5^	8.97 × 10^4^	**1.56 × 10^2^**	**4.87 × 10^1^**	6.94 × 10^3^	6.14 × 10^3^	1.63 × 10^3^	1.31 × 10^3^	3.82 × 10^6^	2.86 × 10^6^	1.59 × 10^3^	2.13 × 10^3^	8.93 × 10^2^	7.10 × 10^2^
F15	4.05 × 10^3^	3.90 × 10^3^	**2.32 × 10^2^**	**8.48 × 10^1^**	1.40 × 10^4^	1.27 × 10^4^	6.67 × 10^3^	2.79 × 10^3^	1.11 × 10^9^	6.72 × 10^8^	2.98 × 10^4^	1.75 × 10^4^	1.41 × 10^4^	3.69 × 10^3^
F16	6.00 × 10^2^	**1.13 × 10^2^**	5.10 × 10^2^	2.47 × 10^2^	9.83 × 10^2^	3.23 × 10^2^	**3.77 × 10^2^**	1.23 × 10^2^	4.76 × 10^3^	1.08 × 10^3^	6.59 × 10^2^	2.70 × 10^2^	5.57 × 10^2^	1.27 × 10^2^
F17	1.93 × 10^2^	4.68 × 10^1^	2.08 × 10^2^	1.57 × 10^2^	4.01 × 10^2^	2.51 × 10^2^	1.46 × 10^2^	**3.87 × 10^1^**	4.14 × 10^3^	3.16 × 10^3^	1.80 × 10^2^	8.00 × 10^1^	**1.16 × 10^2^**	4.47 × 10^1^
F18	2.99 × 10^5^	9.48 × 10^4^	**1.62 × 10^2^**	**6.50 × 10^1^**	1.62 × 10^5^	1.46 × 10^5^	3.20 × 10^4^	2.05 × 10^4^	5.51 × 10^7^	4.16 × 10^7^	7.32 × 10^4^	5.11 × 10^4^	6.63 × 10^4^	2.59 × 10^4^
F19	9.39 × 10^3^	7.09 × 10^3^	**9.86 × 10^1^**	**2.37 × 10^1^**	1.08 × 10^4^	9.59 × 10^3^	7.21 × 10^3^	6.34 × 10^3^	9.91 × 10^8^	4.81 × 10^8^	2.69 × 10^4^	1.48 × 10^4^	1.08 × 10^5^	3.64 × 10^4^
F20	2.48 × 10^2^	6.46 × 10^1^	1.20 × 10^3^	2.02 × 10^2^	3.97 × 10^2^	1.84 × 10^2^	**2.24 × 10^2^**	5.49 × 10^1^	1.07 × 10^3^	1.66 × 10^2^	2.86 × 10^2^	8.96 × 10^1^	2.38 × 10^2^	**5.45 × 10^1^**
F21	2.01 × 10^2^	7.66 × 10^1^	2.33 × 10^2^	8.79	3.28 × 10^2^	3.22 × 10^1^	**1.50 × 10^2^**	**4.29 × 10^−13^**	6.79 × 10^2^	5.28 × 10^1^	2.51 × 10^2^	1.03 × 10^1^	2.26 × 10^2^	7.77
F22	1.10 × 10^2^	7.44	3.59 × 10^3^	2.34 × 10^3^	2.98 × 10^3^	1.80 × 10^3^	1.50 × 10^2^	**0.00**	7.55 × 10^3^	6.27 × 10^2^	2.38 × 10^2^	7.50 × 10^2^	**1.00 × 10^2^**	1.01 × 10^−5^
F23	4.20 × 10^2^	1.90 × 10^1^	1.57 × 10^3^	8.19 × 10^2^	5.06 × 10^2^	5.35 × 10^1^	6.08 × 10^2^	3.41 × 10^1^	1.26 × 10^3^	1.57 × 10^2^	4.13 × 10^2^	1.73 × 10^1^	**3.78 × 10^2^**	**7.74**
F24	3.29 × 10^2^	1.91 × 10^2^	4.42 × 10^2^	**5.87**	5.77 × 10^2^	4.68 × 10^1^	**2.87 × 10^2^**	3.32 × 10^1^	1.44 × 10^3^	1.94 × 10^2^	4.78 × 10^2^	2.31 × 10^1^	4.41 × 10^2^	7.76
F25	**3.84 × 10^2^**	**4.64 × 10^−1^**	3.87 × 10^2^	6.21 × 10^−1^	3.90 × 10^2^	1.02 × 10^1^	4.05 × 10^2^	1.18 × 10^1^	2.64 × 10^3^	5.64 × 10^2^	4.20 × 10^2^	2.49 × 10^1^	3.87 × 10^2^	2.72
F26	3.04 × 10^2^	3.86 × 10^1^	**2.83 × 10^2^**	**3.79 × 10^1^**	2.43 × 10^3^	6.88 × 10^2^	3.68 × 10^2^	4.22 × 10^2^	9.44 × 10^3^	1.14 × 10^3^	1.53 × 10^3^	8.48 × 10^2^	1.25 × 10^3^	3.02 × 10^2^
F27	**5.13 × 10^2^**	**4.55**	1.03 × 10^3^	1.14 × 10^3^	5.53 × 10^2^	2.61 × 10^1^	1.10 × 10^3^	9.91 × 10^1^	1.95 × 10^3^	4.55 × 10^2^	5.65 × 10^2^	2.55 × 10^1^	5.23 × 10^2^	8.67
F28	3.94 × 10^2^	**1.12 × 10^1^**	3.30 × 10^2^	5.18 × 10^1^	3.71 × 10^2^	6.21 × 10^1^	4.11 × 10^2^	2.32 × 10^1^	4.83 × 10^3^	5.65 × 10^2^	3.28 × 10^2^	4.72 × 10^1^	**3.11 × 10^2^**	1.50 × 10^1^
F29	6.33 × 10^2^	6.89 × 10^1^	6.63 × 10^2^	1.40 × 10^2^	1.02 × 10^3^	2.31 × 10^2^	6.32 × 10^2^	7.10 × 10^1^	5.87 × 10^3^	2.02 × 10^3^	8.72 × 10^2^	1.70 × 10^2^	**6.08 × 10^2^**	**5.23 × 10^1^**
F30	1.55 × 10^4^	5.45 × 10^3^	**2.39 × 10^3^**	**6.14 × 10^2^**	1.17 × 10^5^	7.01 × 10^4^	2.28 × 10^5^	1.18 × 10^5^	1.58 × 10^9^	1.05 × 10^9^	8.99 × 10^4^	4.74 × 10^4^	5.11 × 10^5^	1.54 × 10^5^

The bold mark indicates that they are the best results among the algorithms.

**Table 8:** Comparison of function rankings between DSF-ELC and six famous optimization algorithms (D=10).

Fun.	ABC		CMAES		dynFWA		LOTFWA		SSA		SPSO2011		DSF-ELC	
	Mean	Std	Mean	Std	Mean	Std	Mean	Std	Mean	Std	Mean	Std	Mean	Std
F1	4	4	1	1	6	6	2	2	7	7	5	5	3	3
F2	3	3	1	1	5	5	4	4	7	7	2	2	6	6
F3	6	7	1	1	3	3	4	4	7	6	1	1	5	5
F4	3	5	1	1	4	4	5	6	7	7	2	2	6	3
F5	3	1	7	7	5	5	4	4	6	6	2	3	1	2
F6	1	1	7	6	3	4	2	2	6	7	5	5	4	3
F7	3	1	6	7	5	5	4	4	7	6	2	3	1	2
F8	3	2	6	5	5	6	4	3	7	7	2	4	1	1
F9	4	4	7	6	1	1	2	2	6	7	5	5	3	3
F10	1	1	7	7	5	5	2	2	6	3	4	6	3	4
F11	1	1	5	6	4	4	2	3	7	7	6	5	3	2
F12	5	4	1	1	3	3	4	5	7	7	2	2	6	6
F13	2	3	1	1	6	6	3	2	7	7	4	5	5	4
F14	7	7	2	1	1	3	4	5	5	4	3	2	6	6
F15	3	3	1	1	2	4	4	2	7	7	5	5	6	6
F16	3	3	6	7	5	5	1	1	7	6	4	4	2	2
F17	1	1	7	7	4	5	2	2	6	6	5	4	3	3
F18	2	2	1	1	6	6	3	3	7	7	5	5	4	4
F19	4	4	1	1	5	6	2	2	7	7	3	3	6	5
F20	1	1	7	6	2	5	5	4	6	7	4	2	3	3
F21	3	4	6	3	4	7	1	1	7	6	5	5	2	2
F22	2	3	5	1	6	6	1	4	7	7	4	2	3	5
F23	1	6	7	7	4	3	5	5	6	4	3	2	2	1
F24	1	1	3	2	6	6	2	3	7	4	5	5	4	7
F25	1	6	6	2	4	5	2	1	7	7	5	4	3	3
F26	1	5	4	3	3	4	2	6	7	7	5	2	6	1
F27	2	2	5	5	4	4	6	6	7	7	3	3	1	1
F28	1	3	6	4	4	6	3	1	7	7	5	5	2	2
F29	1	5	5	4	6	6	2	2	7	7	4	3	3	1
F30	3	4	1	1	5	5	2	2	7	7	6	6	4	3

**Table 9:** Comparison of function rankings between DSF-ELC and six famous optimization algorithms (D=30).

Fun.	ABC		CMAES		dynFWA		LOTFWA		SSA		SPSO2011		DSF-ELC	
	Mean	Std	Mean	Std	Mean	Std	Mean	Std	Mean	Std	Mean	Std	Mean	Std
F1	3	3	1	1	5	6	2	2	7	7	6	5	4	4
F2	6	6	1	1	3	3	5	5	7	7	2	2	4	4
F3	7	7	1	1	3	3	4	4	6	6	1	1	5	5
F4	1	3	2	2	5	1	3	4	7	7	4	6	6	5
F5	4	2	6	5	5	7	2	3	7	6	3	4	1	1
F6	1	1	6	4	5	7	2	2	7	6	4	5	3	3
F7	4	3	6	7	5	6	2	2	7	5	3	4	1	1
F8	4	5	6	2	5	7	3	3	7	6	2	4	1	1
F9	4	5	6	3	5	6	1	1	7	7	3	4	2	2
F10	2	1	6	2	4	6	3	5	7	4	5	7	1	3
F11	6	6	3	2	5	5	4	4	7	7	2	3	1	1
F12	5	4	1	1	4	5	6	6	7	7	2	2	3	3
F13	3	4	1	1	5	5	2	2	7	7	6	6	4	3
F14	6	6	1	1	5	5	4	3	7	7	3	4	2	2
F15	2	4	1	1	4	5	3	2	7	7	6	6	5	3
F16	4	1	2	4	6	6	1	2	7	7	5	5	3	3
F17	4	3	5	5	6	6	2	1	7	7	3	4	1	2
F18	6	5	1	1	5	6	2	2	7	7	4	4	3	3
F19	3	3	1	1	4	4	2	2	7	7	5	5	6	6
F20	3	3	7	7	5	6	1	2	6	5	4	4	2	1
F21	2	7	4	3	6	5	1	1	7	6	5	4	3	2
F22	2	3	6	7	5	6	3	1	7	4	4	5	1	2
F23	3	3	7	7	4	5	5	4	6	6	2	2	1	1
F24	2	6	4	1	6	5	1	4	7	7	5	3	3	2
F25	1	1	2	2	4	4	5	5	7	7	6	6	3	3
F26	2	2	1	1	6	5	3	4	7	7	5	6	4	3
F27	1	1	5	7	3	4	6	5	7	6	4	3	2	2
F28	5	1	3	5	4	6	6	3	7	7	2	4	1	2
F29	3	2	4	4	6	6	2	3	7	7	5	5	1	1
F30	2	2	1	1	4	4	5	5	7	7	3	3	6	6

**Table 10:** Comparison of function average rankings between DSF-ELC and six famous optimization algorithms (D=10).

Fun.	ABC		CMAES		dynFWA		LOTFWA		SSA		SPSO2011		DSF-ELC	
	Mean	Std	Mean	Std	Mean	Std	Mean	Std	Mean	Std	Mean	Std	Mean	Std
F1–3	4.33	4.67	**1.00**	**1.00**	4.67	4.67	3.33	3.33	7.00	6.67	2.67	2.67	4.67	4.67
F4–10	**2.57**	**2.14**	5.86	5.57	4.00	4.29	3.29	3.29	6.43	6.14	3.14	4.00	2.71	2.57
F11–20	**2.90**	**2.90**	3.20	3.20	3.80	4.70	3.00	2.90	6.60	6.50	4.10	3.70	4.40	4.10
F21–30	**1.60**	3.90	4.80	3.20	4.60	5.20	2.60	3.10	6.90	6.30	4.50	3.70	3.00	**2.60**
F1–30	**2.53**	3.23	4.13	3.53	4.20	4.77	2.97	**3.10**	6.70	6.37	3.87	3.67	3.57	3.30
Best/2nd Best/Worst	11/4/1	8/3/2	10/1/7	12/2/6	2/2/0	1/0/1	3/12/0	4/10/0	0/0/22	0/0/20	1/6/0	1/8/0	4/4/0	5/6/1

The bold mark indicates that they are the best results among the algorithms.

**Table 11:** Comparison of function average rankings between DSF-ELC and six famous optimization algorithms (D=30).

Fun.	ABC		CMAES		dynFWA		LOTFWA		SSA		SPSO2011		DSF-ELC	
	Mean	Std	Mean	Std	Mean	Std	Mean	Std	Mean	Std	Mean	Std	Mean	Std
F1–3	5.33	5.33	**1.00**	**1.00**	3.67	4.00	3.67	3.67	6.67	6.67	3.00	2.67	4.33	4.33
F4–10	2.86	2.86	5.43	3.57	4.86	5.71	2.29	2.86	7.00	5.86	3.43	4.86	**2.14**	**2.29**
F11–20	4.20	3.90	**2.30**	**2.40**	4.90	5.30	2.70	2.60	6.90	6.80	4.00	4.30	3.00	2.70
F21–30	**2.30**	2.80	3.70	3.80	4.80	5.00	3.70	3.50	6.90	6.40	4.10	4.10	2.50	**2.40**
F1–30	3.20	3.30	3.20	2.83	4.40	4.83	2.80	2.80	6.43	5.97	3.53	3.93	**2.53**	**2.43**
Best/2nd Best/Worst	4/7/1	6/4/2	11/3/2	12/5/5	0/0/0	1/0/3	5/9/0	4/9/0	0/0/27	0/0/19	1/6/0	1/3/1	10/4/0	7/8/0

The bold mark indicates that they are the best results among the algorithms.

**Table 12:** Wilcoxon signed rank test results with confidence level of 95% on the CEC 2017 30D problem set. Sum represents the sum of “+”, “−”, and “≈”.

Fun.	vs. ABC		vs. CMAES		vs. dynFWA		vs. LOTFWA		vs. SSA		vs. SPSO2011	
	*p*-Value	Win	*p*-Value	Win	*p*-Value	Win	*p*-Value	Win	*p*-Value	Win	*p*-Value	Win
F1	4.82 × 10^−10^	−	9.43 × 10^−13^	−	2.57 × 10^−1^	≈	1.08 × 10^−10^	−	2.07 × 10^−11^	+	8.80 × 10^−1^	≈
F2	5.37 × 10^−9^	+	9.43 × 10^−13^	−	1.57 × 10^−10^	−	2.03 × 10^−4^	+	2.07 × 10^−11^	+	2.07 × 10^−11^	−
F3	2.07 × 10^−11^	+	9.43 × 10^−13^	−	2.06 × 10^−11^	−	2.07 × 10^−11^	−	2.07 × 10^−11^	+	9.43 × 10^−13^	−
F4	9.16 × 10^−6^	−	9.00 × 10^−7^	−	3.99 × 10^−1^	≈	1.14 × 10^−3^	−	2.07 × 10^−11^	+	2.78 × 10^−2^	−
F5	2.07 × 10^−11^	+	2.06 × 10^−11^	+	2.07 × 10^−11^	+	4.53 × 10^−11^	+	2.07 × 10^−11^	+	4.11 × 10^−11^	+
F6	9.43 × 10^−13^	−	2.07 × 10^−11^	+	2.07 × 10^−11^	+	4.14 × 10^−9^	−	2.07 × 10^−11^	+	2.07 × 10^−11^	+
F7	2.07 × 10^−11^	+	2.09 × 10^−10^	+	2.07 × 10^−11^	+	3.15 × 10^−7^	+	2.07 × 10^−11^	+	1.08 × 10^−10^	+
F8	2.07 × 10^−11^	+	1.92 × 10^−11^	+	4.53 × 10^−11^	+	2.07 × 10^−11^	+	2.07 × 10^−11^	+	1.57 × 10^−10^	+
F9	2.07 × 10^−11^	+	2.07 × 10^−11^	+	2.07 × 10^−11^	+	2.15 × 10^−11^	−	2.07 × 10^−11^	+	1.43 × 10^−9^	+
F10	3.60 × 10^−1^	≈	2.03 × 10^−11^	+	3.03 × 10^−10^	+	3.10 × 10^−2^	−	2.07 × 10^−11^	+	7.61 × 10^−10^	+
F11	2.07 × 10^−11^	+	1.61 × 10^−6^	+	9.13 × 10^−10^	+	5.77 × 10^−7^	+	2.07 × 10^−11^	+	2.32 × 10^−7^	+
F12	6.38 × 10^−2^	≈	2.07 × 10^−11^	−	8.01 × 10^−1^	≈	2.67 × 10^−9^	+	2.07 × 10^−11^	+	4.97 × 10^−6^	−
F13	4.06 × 10^−5^	−	2.07 × 10^−11^	−	9.71 × 10^−1^	≈	4.82 × 10^−10^	−	2.07 × 10^−11^	+	1.16 × 10^−8^	+
F14	2.07 × 10^−11^	+	5.50 × 10^−11^	−	1.26 × 10^−8^	+	5.25 × 10^−3^	+	2.07 × 10^−11^	+	1.47 × 10^−1^	≈
F15	4.82 × 10^−10^	−	2.07 × 10^−11^	−	5.12 × 10^−1^	≈	1.43 × 10^−9^	−	2.07 × 10^−11^	+	6.25 × 10^−5^	+
F16	1.05 × 10^−1^	≈	5.21 × 10^−1^	≈	2.50 × 10^−7^	+	3.52 × 10^−6^	−	2.07 × 10^−11^	+	1.39 × 10^−1^	≈
F17	3.67 × 10^−7^	+	5.98 × 10^−2^	≈	4.04 × 10^−8^	+	3.82 × 10^−3^	+	2.07 × 10^−11^	+	7.56 × 10^−4^	+
F18	2.28 × 10^−11^	+	2.07 × 10^−11^	−	1.36 × 10^−4^	+	2.15 × 10^−7^	−	2.07 × 10^−11^	+	9.71 × 10^−1^	≈
F19	2.28 × 10^−11^	−	2.07 × 10^−11^	−	2.28 × 10^−11^	−	2.07 × 10^−11^	−	2.07 × 10^−11^	+	8.90 × 10^−11^	−
F20	8.91 × 10^−1^	≈	2.07 × 10^−11^	+	4.59 × 10^−5^	+	2.07 × 10^−1^	≈	2.07 × 10^−11^	+	1.91 × 10^−2^	+
F21	1.82 × 10^−1^	≈	9.79 × 10^−4^	+	2.07 × 10^−11^	+	9.43 × 10^−13^	−	2.07 × 10^−11^	+	1.57 × 10^−10^	+
F22	4.00 × 10^−10^	+	7.35 × 10^−3^	+	3.53 × 10^−4^	+	9.41 × 10^−13^	+	2.07 × 10^−11^	+	2.42 × 10^−4^	+
F23	2.28 × 10^−11^	+	6.22 × 10^−7^	+	2.07 × 10^−11^	+	2.07 × 10^−11^	+	2.07 × 10^−11^	+	2.52 × 10^−10^	+
F24	2.58 × 10^−2^	−	5.79 × 10^−1^	≈	2.07 × 10^−11^	+	3.03 × 10^−12^	−	2.07 × 10^−11^	+	3.33 × 10^−10^	+
F25	1.30 × 10^−10^	−	4.40 × 10^−1^	≈	2.31 × 10^−6^	+	1.43 × 10^−10^	+	2.07 × 10^−11^	+	1.57 × 10^−9^	+
F26	4.51 × 10^−9^	−	5.71 × 10^−10^	−	4.51 × 10^−9^	+	3.08 × 10^−8^	−	2.07 × 10^−11^	+	2.27 × 10^−3^	+
F27	1.73 × 10^−6^	−	2.06 × 10^−2^	+	3.16 × 10^−8^	+	2.07 × 10^−11^	+	2.07 × 10^−11^	+	1.30 × 10^−10^	+
F28	2.52 × 10^−11^	+	1.38 × 10^−3^	+	7.02 × 10^−2^	≈	2.07 × 10^−11^	+	2.07 × 10^−11^	+	1.43 × 10^−3^	+
F29	6.17 × 10^−2^	≈	2.57 × 10^−1^	≈	6.95 × 10^−10^	+	1.32 × 10^−1^	≈	2.07 × 10^−11^	+	2.05 × 10^−9^	+
F30	2.07 × 10^−11^	−	2.07 × 10^−11^	−	2.52 × 10^−11^	−	3.79 × 10^−9^	−	2.07 × 10^−11^	+	2.07 × 10^−11^	−
sum	13/11/6		13/12/5		20/4/6		13/15/2		30/0/0		20/6/4	

## Data Availability

The original contributions presented in this study are included in the article. Further inquiries can be directed to the corresponding author.
